# Data cleaning and harmonization of clinical trial data: Medication-assisted treatment for opioid use disorder

**DOI:** 10.1371/journal.pone.0312695

**Published:** 2024-11-21

**Authors:** Raymond R. Balise, Mei-Chen Hu, Anna R. Calderon, Gabriel J. Odom, Laura Brandt, Sean X. Luo, Daniel J. Feaster

**Affiliations:** 1 Department of Public Health Sciences, University of Miami Miller School of Medicine, Miami, Florida, United States of America; 2 Department of Psychiatry, Vagelos College of Physicians and Surgeons, Columbia University, New York, New York, United States of America; 3 Department of Biostatistics, Stempel College of Public Health, Florida International University, Miami, Florida, United States of America; 4 Department of Psychology, City College of New York, New York, New York, United States of America; University of Lucknow, INDIA

## Abstract

Several large-scale, pragmatic clinical trials on opioid use disorder (OUD) have been completed in the National Drug Abuse Treatment Clinical Trials Network (CTN). However, the resulting data have not been harmonized between the studies to compare the patient characteristics. This paper provides lessons learned from a large-scale harmonization process that are critical for all biomedical researchers collecting new data and those tasked with combining datasets. We harmonized data from multiple domains from CTN-0027 (N = 1269), which compared methadone and buprenorphine at federally licensed methadone treatment programs; CTN-0030 (N = 653), which recruited patients who used predominantly prescription opioids and were treated with buprenorphine; and CTN-0051 (N = 570), which compared buprenorphine and extended-release naltrexone (XR-NTX) and recruited from inpatient treatment facilities. Patient-level data were harmonized and a total of 23 database tables, with meticulous documentation, covering more than 110 variables, along with three tables with “meta-data” about the study design and treatment arms, were created. Domains included: social and demographic characteristics, medical and psychiatric history, self-reported drug use details and urine drug screening results, withdrawal, and treatment drug details. Here, we summarize the numerous issues with the organization and fidelity of the publicly available data which were noted and resolved, and present results on patient characteristics across the three trials and the harmonized domains, respectively. A systematic harmonization of OUD clinical trial data can be accomplished, despite heterogeneous data coding and classification procedures, by standardizing commonly assessed characteristics. Similar methods, embracing database normalization and/or “tidy” data, should be used for future datasets in other substance use disorder clinical trials.

## Introduction

Biostatisticians, data scientists, and clinical investigators long for the ability to apply machine learning algorithms to make “high stakes”, often life-or-death, predictions, but these efforts are impeded by a lack of clean, harmonized, clinically relevant data [1]. A germane example is predicting response to treatment for opioid use disorder (OUD). With an annual death toll that hovered near 50,000 Americans in the late 2010s and a rising count likely to hit 100,000 deaths in 2022, misuse of opioids has led to staggering levels of human suffering in the United States [2]. The weight of these numbers is amplified when one realizes there are efficacious medical treatments for OUD [3–5]. However, while these pharmacological treatments can work, matching individuals to the right medications at the correct dosages and in the correct settings (i.e., inpatient vs. outpatient) so that patients can complete treatment and work toward long term recovery has proven to be extraordinarily difficult for clinicians and clinical researchers [3–6].

Progress in OUD treatment research is hampered by the lack of a central repository for harmonized, individual-level data gathered during treatment trials. This lack of publicly accessible information prevents the review and independent analysis of these data by researchers and clinicians who were not directly involved in the primary trials.

Computer and data scientists have published extensively on the harmonization process in many domains and there are well established systems, such as the Observational Medical Outcomes Partnership (OMOP; https://ohdsi.github.io/CommonDataModel/). However, a 2024 review published in Nature points out that this guidance is highly technical and domain-specific [7]. The arguably most relevant guidance for substance use disorder researchers, the Maelstrom Research guidelines, were built from dozens of international research initiatives but do not include any representation from the field of addiction research [8–10].

The reason for the lack of a central repository for individual participant-level data from substance use disorder (SUD) clinical trials is many-fold. The designs of these trials are heterogeneous. Even within the domain of OUD, there are different medications, psychotherapy programs, and types of substances (e.g., heroin vs. prescription opioids). Clinical trials use different assessment instruments, which are updated frequently, and outcome measures are made over different lengths of time. Additionally, while many funding agencies have stipulated uniform policies for data sharing and dissemination, many investigators do not publicly release their datasets. Pivotal trial data for many of the commercial medications for opioid use disorder (MOUD) treatments remain largely proprietary [11].

Expectations of transparency in data reporting in clinical trials are becoming universal: initiatives by the European Medicines Agency and the National Academies of Sciences, Engineering and Medicine (formerly Institute of Medicine) have promulgated rough guidelines. Nevertheless, standardization in clinical trial data sharing continues to be challenging and is an active research agenda in biomedical informatics. Standardization efforts have yielded valuable data sources in several areas of medicine such as oncology and cardiovascular medicine [12–14]. In addiction medicine, such efforts remain in their infancy. Under the umbrella of the National Institute on Drug Abuse (NIDA), the National Drug Abuse Treatment Clinical Trials Network (CTN) created a multi-institutional network of government-funded clinical research sites to carry out SUD treatment trials [15]. Data from these studies are available on the NIDA Data Share website [16]. While there are widely used standards for structuring and sharing data in the medical domain, such as the Observational Medical Outcomes Partnership (OMOP) Common Data Model (CDM) with its Observational Health Data Sciences and Informatics (OHDSI) tools [17], relatively little rigorous advice has been offered on the harmonization process itself [9]. To date, the NIDA Data Share repositories have not been harmonized. The only attempts to standardize responses to self-reported drug use questionnaires have led to a few tailored common data elements (CDEs), but fundamental details needed to harmonize data on people getting care for OUD are missing. For example, there are no CDEs for sex assigned at birth or urine drug screening results. Further, many CDEs are missing details such as “refused to answer” or “unknown”[16, 18]. So, investigators have been unable to assess the similarities and differences in treatment-seeking individuals across trials. A prior attempt to harmonize data contained in the NIDA Data Share neither provided concrete advice on data cleaning nor adopted commonly accepted guidance on variable or table names, such as the Tidyverse style guide, and it also did not follow accepted best practices for database design, such as removing unnecessary column redundancy [19]. Furthermore, the team did not publish a detailed schema describing the data tables or variables, which hinders the replication of this harmonization effort and limits the use of the (non-released) harmonized database for understanding the landscape of care-seeking individuals and their responses to treatment for substance use disorders. The CTN-0094 project aimed to address these shortcomings by implementing a rigorous and transparent approach to harmonizing data, focusing specifically on large OUD clinical trials, and making the resulting dataset freely accessible to interested stakeholders.

The goal of CTN-0094 was to use data from the three largest pragmatic trials on MOUD] to understand who responds to which treatments for OUD and to build statistical models to help match care-seeking individuals to their optimal medication [5, 20–23]. This is the first study to attempt comprehensive harmonization of CTN data specifically targeting OUD clinical trials.

This paper presents details on the data cleaning and the harmonization process for CTN-0094. It provides lessons learned that are critical not only for individuals interested in the treatment of SUDs but all biomedical researchers collecting new data and those tasked with combining datasets. Our goal here is two-fold. We hope to leave the reader with a clear understanding of the individuals who received care in the trials that fed CTN-0094, thus offering other research teams the opportunity to build models to predict treatment success for people suffering from OUD. The janitorial work on these data took an unexpectedly large amount of time. Therefore, our second goal is to provide a list of guidance and checks that should be in place for any data collection system used to track clinical data.

## Methods

### Source data studies

This study harmonized existent, deidentified data which was locked at the completion of each of three multisite trials for the treatment of OUD: Starting Treatment with Agonist Replacement Therapies (START, CTN-0027) [20], Prescription Opioid Addiction Treatment Study (POATS, CTN-0030) [21] and Extended-Release Naltrexone (XR-NTX) vs. Buprenorphine (BUP-NX) for Opioid Treatment (X:BOT, CTN-0051) [22]. Following approval by the New York State Psychiatric Institute—Columbia University Department of Psychiatry Institutional Review Board, these frozen datasets were accessed between October 2019 and October 2023. All studies involved treatment-seeking individuals with either DSM-IV-defined opioid dependence (CTN-0027 and CTN-0030) or DSM-5-defined OUD recruited from federally licensed outpatient (CTN-0027 and CTN-0030) or inpatient treatment facilities (CTN-0051), and they were in treatment for 24 weeks. CTN-0027 participants were treated with either buprenorphine/naloxone (BUP) or methadone delivered daily. The primary outcome was liver toxicity at 24 weeks. For CTN-0030, treatment was delivered in two phases. In the first Phase, participants received either BUP and standard medical management for two weeks and a subsequent taper of BUP for two weeks *or* enhanced counseling and Standard Medical Management (SMM) with BUP for two weeks and a subsequent taper of BUP for two weeks. All participants were followed for an additional eight weeks to assess treatment success. Participants considered “treatment failures” in Phase 1 were rerandomized in Phase 2 to a similar protocol, but the active BUP lasted for 12 weeks, and a subsequent taper for four weeks; there were eight follow-up weeks to assess the outcomes of Phase 2. For CTN-0051, the treatment consisted of 24 weeks of XR-NTX, which was delivered every four weeks as intramuscular injections, or BUP-NX, which were self-administered daily. Details on the study interventions can be found in the source articles listed above.

### Sample size and study population

**Fig 1:**
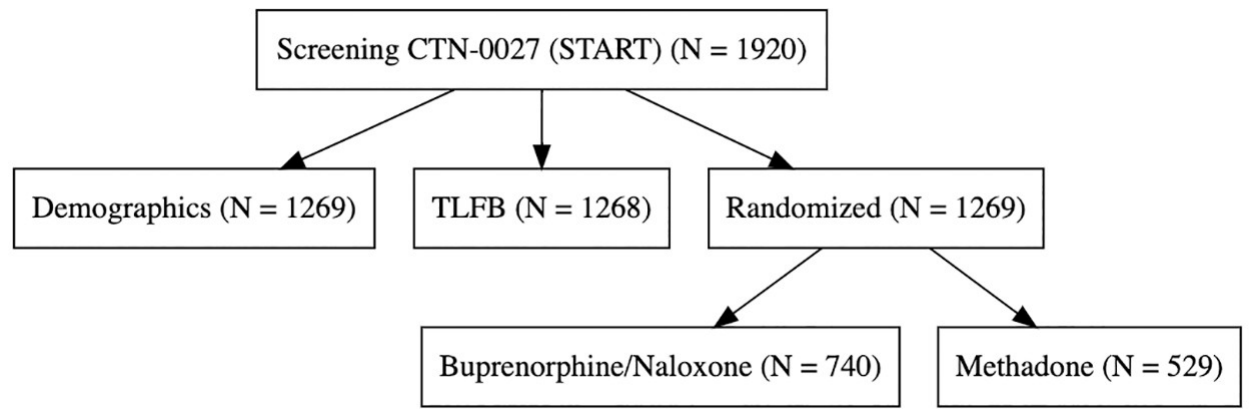
Sample size for CTN-0027. Illustration of Source Data.

**Fig 2:**
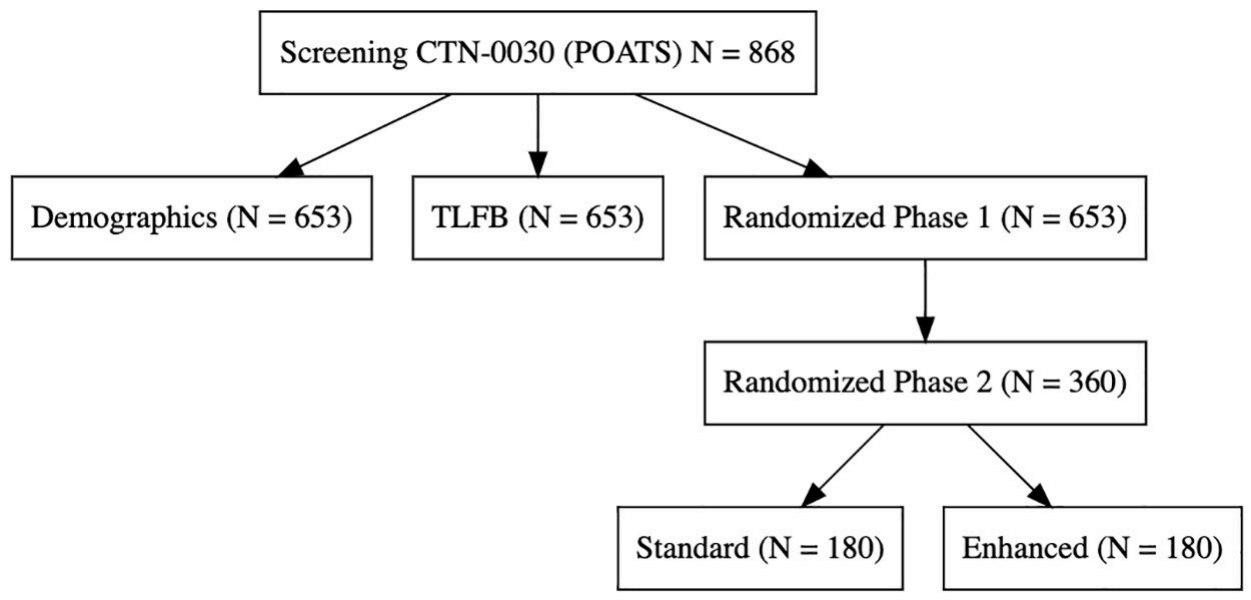
Sample size for CTN-0030. Illustration of Source Data.

**Fig 3:**
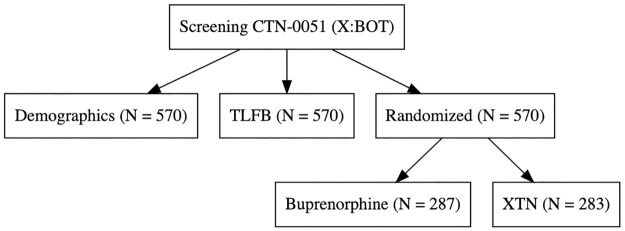
Sample size for CTN-0051. Illustration of Source Data.

The harmonized data contains data on 3560 people. While analyses studying treatment efficacy will be restricted to randomized individuals, many individuals were screened but never randomized (N = 1068), and their baseline data could also be harmonized to create a larger baseline cohort (Figs 1–3). Some of these people (N = 45) have the combination of both drug use information and demographics, which affords additional information on polysubstance drug use in people awaiting MOUD care. Figs 2 and 3.

In CTN-0027, 1269 participants were randomized, with 740 assigned to BUP and 529 to MET, with a large proportion of individuals who remained in treatment (52% (N = 387) for BUP and 77% (N = 410) for MET) versus individuals who missed more than 14 days of treatment (353 for BUP and 119 for MET). For CTN-0030, 653 individuals were randomized in Phase 1, with 329 to Opioid Dependence Counseling (ODC) plus Standard Medication Management (SMM) and 324 to SMM alone in a two-phase adaptive design; hence, they can be thought of as either having BUP alone or BUP plus a specific, enhanced psychotherapy program. In CTN-0051, 570 individuals were randomized from inpatient detoxification and for their first treatment, 283 were randomized to XR-NTX and 287 randomized to BUP and all were followed as outpatients. While the three studies aimed to recruit individuals who presented in different contexts, all three studies had at least six months of planned follow-up: standardized follow-up schedules, urine toxicology data, and overlapping standardized baseline assessment instruments.

### Survey domains and harmonization techniques

The CTN-0094 team, which includes domain experts in data science, biostatistics, psychology, and psychiatry, all with experience in clinical trials and OUD treatment, reviewed all items of the Case Report Forms (CRFs) and survey schedule tables from the three studies. Following guidance offered in the Maelstrom Research guidelines for data harmonization, a large, inclusive database table was constructed to comprehensively document all the overlapping questions/domains, with well over 1,500 individual items surveyed [9]. A subset of the CRFs has conceptually similar/equivalent items. From this, a set of database tables were designed. These follow a relational model, meaning they can be joined together using the key variables “who” (i.e., subject ID number). Each table represents a “domain” or “topic” including 1) sociodemographic features including age, gender/sex, race/ethnicity; 2) self-reported substance use captured with the Timeline Followback (TLFB) method of assessment urine drug screening (UDS); 4) nicotine use; 5) opioid withdrawal symptoms; 6) medical and psychiatric history; 7) quality of life; 8) risky behaviors related to drug use; 9) randomization to study conditions; 10) treatment and medication doses; and 11) dates of trial design-specific events and assessments [24].

We aligned variables from the three trials and standardized their values when their coding was unambiguous (e.g., sex assigned at birth). Other variables were semantically very similar but not identical, so they needed to be reported as two variables. For example, the urine drug screening reported concentrations of opioids at 300 ng/mL or 2000 ng/mL. If there were any questions about whether two variables were semantically the same, the data science team consulted with the MDs and PhDs on the CTN-0094 team to resolve questions. In the rare cases where there was still uncertainty, the original study authors were contacted. Similar issues arose when two different measures were used to assess the same construct. For example, withdrawal symptoms for some people were calculated as a numeric score by self-report (i.e., the SOWS in CTN-0051), and others, by a clinician (i.e., the COWS in CTN-0027 and CTN-0030). For these variables, there is no published literature to equate numeric scores (e.g., severe withdrawal) on the two scales. Therefore, there was no obvious path to combine/harmonize these data. The medical board-certified addiction experts on the study team, in consultation with other addiction specialists, helped build the crosswalk between these variables. Similar issues arose with standardizing self-reported pain. The specific details on those decisions are documented in the harmonization vignette (https://cran.r-project.org/web/packages/public.ctn0094data/vignettes/harmonization.html) of the public.ctn0094data package discussed below. That vignette also includes examples of how variables were recoded to support analyses. For example, the missed-visit details have self-reported free text answers like “prison”, “jail” and “incarcerated” which were recategorized as “In Jail”. A general concern was to avoid loss of information while providing tables that would be useful for analysts. As explained in the vignette and discussed below, some redundancy was retained in the database, for example there is a table (i.e., all_drugs) that contains every illicit drug reported from every source and also a table that includes self-reported drugs from the “timeline follow back” interview (i.e., tlfb), grouped into categories that we expect analysts will want.

Anonymized, individual-level investigators’ datasets were obtained from each study data management team. PDF documentation of the study data was provided. Documentation included CRFs suitable for manual data collection, semi-structured PDF code books (with different formats across the trials), and screenshots of data collection forms. The CRFs were recorded variably in the three studies. CTN-0027 and CTN-0030 have a separate table that details the schedule of the surveys and abbreviations, whereas CTN-0051 does not. CTN-0027 and CTN-0030 have an easy-to-read manual of CRFs collected, but CTN-0051 does not. It is important to note that these files, like many PDFs, which are missing tags to indicate the semantic structure of the content and instead store information as strategically positioned lines and text, are not *at all* straightforward for a computer to parse [25]. That is, there were no “meta-data” database tables available, and it was practically impossible to build those tables. Therefore, there was no “computer-readable” documentation covering details on what variables and concepts were assessed in each study, and there was no “computable” information on key details like permitted values for variables. In other words, because of the structure of the documentation, the harmonization process could not be automated. Instead, it required highly time-intensive reading and curating of the documentation and abstraction of the variable names, definitions, and permitted values. Even trivial tasks like defining the sex of participants, which in theory, could be fully automated with look-ups in computer-friendly meta-data tables, required manual review of CRFs. In a few cases, variable code values were printed “off the edge of the page.” Therefore, codes had to be guessed based on context, content, and similar names in other trials. Further, some levels of categorical variables are missing documentation, again forcing guessing. Finally, some data are entirely missing from the documentation. For example, instruments that were initially used but were later changed or dropped, such as the World Health Organization Composite International Diagnostic Interview (CIDI) and the Short Form Health Survey 36 (SF-36) measures initially used in CTN-0027, were excluded from the documentation.

**Fig 4:**
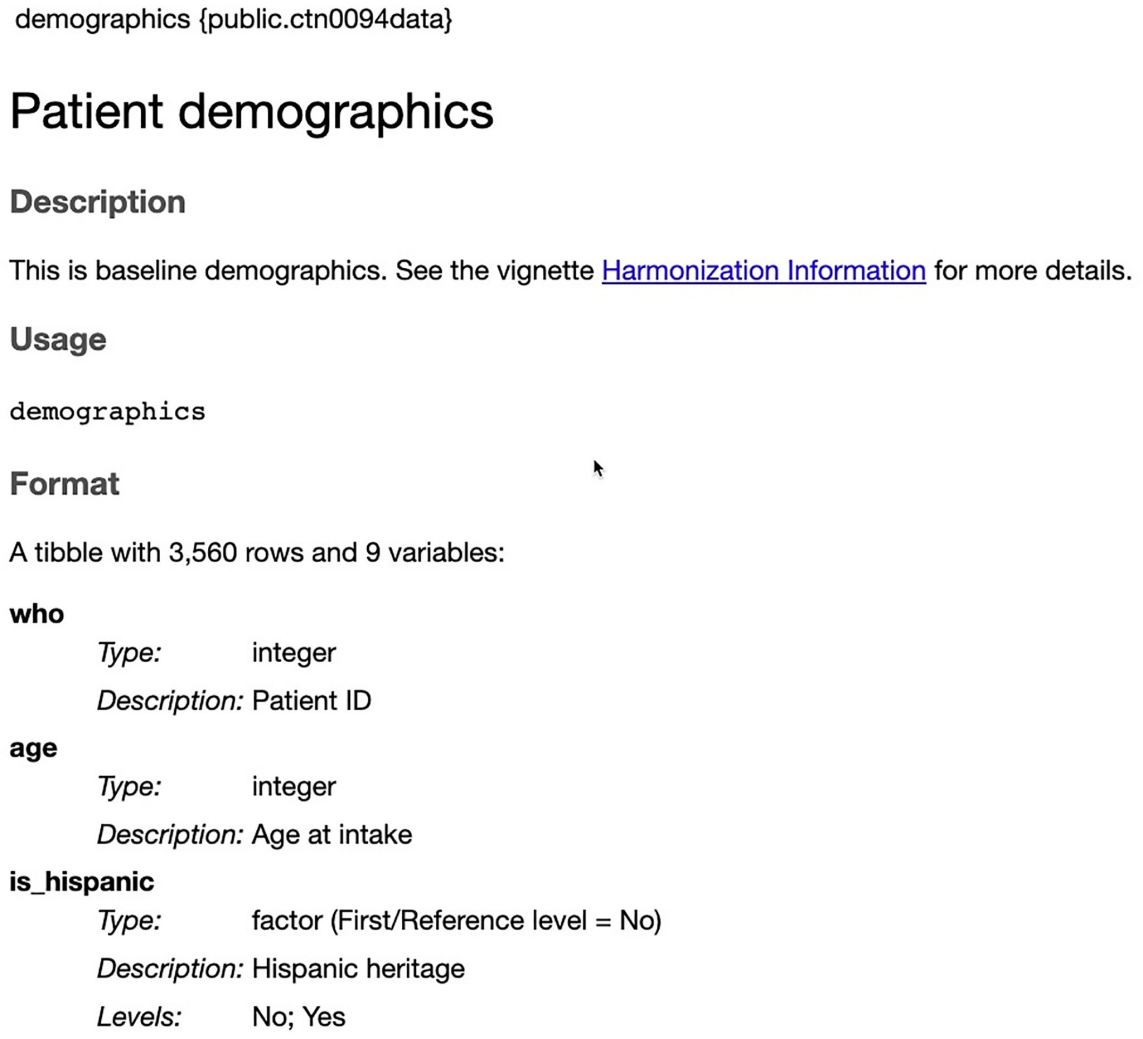
Example documentation. The documentation for the first three variables in the demographics table in the CTN0094data package.

In contrast to the strategy of only providing human-readable PDF documentation, the harmonized data which accompanies this manuscript is available in an R data package named public.ctn0094data. The package has “manual” pages covering the variable names, types, and permitted values in both human-readable web pages and computer-readable, annotated text format. In fact, the human-readable documentation was automatically produced by processing the text files using the roxygen2 (version 7.2.3) [26] and pkgdown (version 2.0.7) [27]. This documentation, an example of which is shown in Fig 4, can be quickly and easily accessed when working in the R language (e.g., by typing? demographics) or on the web (https://ctn-0094.github.io/public.ctn0094data/reference/demographics.html). The documentation shows the name of each variable, its type (i.e., integer or categorical factor), a brief descriptive label, the permitted values, and an indicator of the default reference level if the variable is used as a predictor or outcome in a statistical model. This documentation is organized by topic in a searchable hyperlinked set of pages which allows users to quickly find the details they need to conduct analyses on these data without having to search though every key word match in the traditional PDF documentation.

A novel contribution of this study was a “double helix” approach to the harmonization process, meaning two independent data science teams worked independently and compared results. For example, both teams identified the variables which were common across the studies, such as sex assigned at birth, in the source data, and wrote code to combine the data into a demographics table. Emails were exchanged between the teams many times each week to address simple questions about what numeric codes should be used (i.e., “How are you coding different amounts of education?”) or when possible problems were identified (i.e., “Do you see person 12 having two contradictory urine drug screens on June 24, 1999?”). Weekly meetings with the complete study team were held to address more complicated questions, such as the harmonization of features like withdrawal and pain discussed above. This multiyear process involved each study site independently studying the raw data and finding and documenting problems and proposing fixes. For example, both sites independently wrote code to identify urine drug screening (UDS) date problems (e.g., testing was reportedly administered before the start of the study or years in the future, or when multiple records were reported on the same date without explanation/justification). Figuring out such date errors required the manual review of both the records with the problematic dates and all of the other records for a given person. For such a UDS date problem, the process involved looking at the timestamps on when data was collected, the reported “study week”, and any other dates within the record (e.g., if a date for an alcohol breathalyzer test was reported on that record). Potential fixes involved checking the complete series of records for each person to see if there was a missing record that corresponded to an “off by one error”, shifting to the wrong month or year, or a transposition of digits for the problematic date. When fixing dates for study drug administration, drug doses in the prior and subsequent days were checked. This process was handled through email correspondence and regular study meetings. The entire study team ultimately reviewed ambiguities. The final harmonized dataset was verified by both study CTN-0094 sites independently to ensure uniformity and consistency. The magnitude of these issues is described below.

### Database structure

Patient-level data were harmonized and a total of 23 database tables with more than 110 variables, along with three tables with “meta-data” about the study design and treatment arms, were created. These tables, along with per-table documentation and long-form explanatory vignettes, are available in a R software package called public.ctn0094data. The latest official release can be downloaded from CRAN, or the development version, which contains prereleases of extra documentation and bug fixes, can be downloaded from GitHub. The full package documentation is also available online.

Datasets were normalized to reduce redundancy and improve data integrity [27, 28]. That is, individual concepts/events (e.g., randomization events or UDS visits) were put into their own data tables, and columns were constructed to remove the redundant variables created when daily data was stored as weekly or monthly records. Details on these tables are described below. Each of these database tables was designed in a “tidy”, or normalized, format. The term “tidy” is commonly used within the data science community, whereas “normalized” is popular with the computer science aficionados. Both terms mean that redundancy is removed in data storage and within each table, each concept is stored in a single variable. For example, a self-reported drug use table was constructed with variables named “project” (CTN-0027, CTN-0030 or CTN-0051), “who” (subject ID numbers), “when” (number of days relative to enrollment) and “what” (drug name). This long (i.e., one record per drug used on each day for each person), skinny (i.e., only four variables: project, who, what, and when) format deviated from the raw data that organized the data in many distinct formats, for example, a study could have a variable corresponding to yes/no cocaine use on day 7. While the datasets were designed to approximate a relational database (i.e., using key variables with unique patterns used to link the subjects across the tables), base code in the R language does not have built-in mechanisms to enforce rules on the dependencies across tables nor does it have tools to support referential integrity. This lack of referential integrity means that R does not provide automatic mechanisms to ensure that people in the “treatment drug” table are in the “randomization” table. However, the data were manually curated to detect discrepancies.

While a primary goal of the database table design was to simplify unnecessary complexity and redundancy seen in the not-normalized original datasets, several tables were created that contain somewhat redundant information. For example, a table that has all drugs detected (from both self-report and UDS) is provided (i.e., *all_drugs*) along with a table of just the self-reported drugs (i.e., *tlfb*) and urine drug screening results (i.e., *uds*). The self-report and screening tables have drugs collapsed into categories (i.e., opiates grouped together) to support the analysis of treatment failure. While the *all_drugs* table includes all detected substances, the *uds* table does not include the prescribed treatment drugs that were detected. Further details are described in the Urine Drug Screening and Timeline Follow-Back sections below.

Table and variable names were designed to be self-evident and follow the not-capitalized “snake_case” recommendation in the R Tidyverse Style Guide [29, 30]. Specifically, the variables “who” (containing the subject ID), “when” (containing the days since consent) and “what” (holding drug names) are used throughout. The need for adopting a simple variable naming convention is evidenced by the seven distinct subject identification variables used across the three studies’ documentation and the raw data analysis files (i.e., tcPATNUM, txtPInumber, PATNUM, ID, PROJID, patientnumber, PATID) and the myriad variable names used for dates. Unsurprisingly, variable names used to indicate drug use were inconsistent across studies. Perhaps surprising was the fact that drug variable names within a study were inconsistent across context (e.g., the variable name for the indicator for self-reported cocaine use vs. the variable name for the UDS detection of cocaine) and in some cases, across the day of the week when the substances were used. These inconsistencies resulted from the use of the variable names used by the data collection system, without an automated crosswalk between those variable names and abstract concepts like “who used what, when.” Further details on drug names are discussed below.

Within the public.ctn0094data package, binary indicator variables use names with a verb prefix and are coded with 0 meaning “no” and 1 meaning “yes.” For example, a variable named “is_male” holds the integer 1, meaning yes, this is a male, or 0, meaning no, this is not a male. This convention, designed to lighten the memory load for analysts, contrasts with the ambiguous variable name and coding scheme (e.g., sex was coded 1 or 2) used in the raw data. That design requires analysts to refer back to the documentation to determine what the value 2 means for any project. Further information on each variable is included in each table’s “manual” page in the public.ctn0094data R package. Supplemental tables and plots are included in the package’s “manuscript” vignette. Additional information about the harmonization process, including exacting details such as the harmonized definition of “severe” withdrawal, can be found in the “harmonization” vignette.

Data were initially cleaned and database tables were created using SAS software Version 9.4 (TS Level 1M7) by two senior statistical programmers working independently. The resulting database tables were aggregated into summary tables similar to those shown below, and all discrepancies were resolved with the help of the entire CTN-0094 team. These data were imported, preprocessed and summarized, and inferential statistics were conducted with R version 4.3.1 (2023-06-16) [31] using the following packages: broom (version 1.0.5) [32], DiagrammeR (version 1.0.10) [33], ggthemes (version 4.2.41) [34], haven (version 2.5.3) [35], infer (version 1.0.5) [36], janitor (version 2.2.0) [37], knitr (version 1.44) [38], kableExtra (version 1.3.4) [39], psych (version 2.3.6) [40], rmarkdown (version 2.25) [41], Table 1 (version 1.4.3) [42], tidyverse (version 2.0.0) [43], rUM (version 1.0.2) [44], vcdExtra (version 0.8.5) [45].

**Table 1:** Drugs assessed by structured questions in timeline follow-back questionnaires.

Substance	CTN-0027	CTN-0030	CTN-0051	Drug Category
Alcohol	Yes	Yes	Yes (Standard Drinks)	Alcohol
Amphetamine	Yes	Yes	Yes	Amphetamine
Buprenorphine	No	No	Yes	Buprenorphine
Ecstasy	No	No	Yes	MDMA
Sedative Barbiturates	No	No	Yes	Barbiturate
Sedatives other than Benzodiazepines	No	Yes	No	Sedatives
Benzodiazepines	Yes	Yes	Yes	Benzodiazepine
Cannabinoids (THC)	Yes	Yes	Yes	THC
Cocaine	Yes	Yes	Yes	Cocaine
Crack	No	No	Yes	Cocaine
Inhalants	No	No	Yes	Inhalant
Methadone	No	Yes	Yes	Methadone
Methamphetamine	Yes	Yes	No	Amphetamine
Opiates	Yes	No	No	Heroin
Opioid Analgesics	No	No	Yes	Opioid
Heroin	No	Heroin/Opium	Yes	Heroin
Morphine	No	Yes	No	Opioid
Hydromorphone	No	Yes	No	Opioid
Codeine	No	Yes	No	Opioid
Oxycodone	Yes	Yes	No	Opioid
Hydrocodone	No	Yes	No	Opioid
Propoxyphene	Yes	Yes	No	Opioid
Other Opiates	No	Yes	No	Opioid
Other Drug 1	No	No	Yes	
Other Drug 2	No	No	Yes	
Other Drugs	Yes	No	No	

Here we describe harmonization details and summaries of the harmonized data. Summary tables with measures of central tendency and variability are provided along with graphics showing distributions for not-normally-distributed, continuous variables. Differences between trials were assessed using ANOVA methods with Tukey’s HSD contrasts and chi-square or Fisher’s exact tests with pairwise proportion tests with Holm-adjusted p-values. Given that the large sample size affords the ability to declare trivial differences as statistically significant, discussions of the results focus on large effect sizes.

### Social and demographic characteristics

#### Age

Date of birth was surveyed directly in all three studies. CTN-0051 also recorded the self-reported age at screening. We harmonized an age variable produced by taking the integer portion of the difference in years between the date of birth and the screening survey date. No discrepancies between the two measurements in CTN-0051 were observed.

#### Gender and sex

None of the harmonized studies assessed gender. For CTN-0027 and CTN-0030, sex could be “Male” or “Female”. For CTN-0051, additional options included “Don’t Know” and “Refused”. However, none of the participants gave these answers in the investigators’ datasets. Therefore, we harmonized sex as a variable indicating whether a subject “is male.”

#### Race and ethnicity

For CTN-0027 and CTN-0030, ethnicity was surveyed as either “of Spanish origin, Hispanic or Latino” or “Not of Spanish origin, Hispanic or Latino”. Further stratification options within the Hispanic ethnicity designation included “Mexican”, “Puerto Rican”, “Cuban” and “other”. In CTN-0051, there was a self-identified Hispanic/Latino question corresponding to this item, which was surveyed as “Yes”, “No”, “Don’t Know”, or “Refused”. The options for Hispanic origin included “Puerto Rican”, “Dominican”, “Mexican”, “Mexican American”, “Chicano”, or “Other”. Hispanic ethnicity was collapsed into a binary indicator.

In both CTN-0027 and CTN-0030, race was surveyed as “American Indian or Alaska Native”, “Asian” with follow-up questions for six subgroups plus “other”, “Black or African American”, “Native Hawaiian or Pacific Islander” with three subgroups plus “other”, “White”, “Other”, “Participant Chooses Not to Answer”, or “Unknown”. In CTN-0051, race contains more than 20 categories, including categories detailing various Asian subgroups, other, refused, and unknown. Given the additional complexity for race, we created a harmonized categorical variable for the Race Category (i.e., White, Black, Refused/Missing, and Other). These represent the predominant racial categories currently surveyed by the United States census, thus affording relatively easy comparisons against the American Community Survey Data provided by the Census Bureau.

#### Additional sociodemographic information

Sociodemographic features were gathered as part of the structured baseline interview in all trials and from the ASI-Lite questionnaire [46], which was administered to an initial subset of patients in CTN-0027 and the majority of the participants in CTN-0030 and CTN-0051. While the documentation of the ASI-Lite variables was missing for CTN-0027 and incomplete for CTN-0030, most of the items could be harmonized. It was assumed that variables with the same name from CTN-0027 and CTN-0030 were the same. Variable names and codes were extremely inconsistent between CTN-0030 and CTN-0051. For example, in CTN-0030, the ASI-Lite question “L2” which asks, “Are you on parole or probation?” was given the variable name L2 and the response “Yes, parole or post-release supervision” was coded with the value “2”, whereas the variable name in CTN-0051 was ALPROBAT and the same response was coded as “1”. Observed, coded values were checked across the three studies and discrepancies were harmonized.

CTN-0051 contained a direct survey of the highest grade/level of education and whether the participant was working, unemployed, or retired as part of the baseline demographic assessment. However, CTN-0027 and CTN-0030 did not contain these direct surveys but similar information was included in the ASI-Lite inventory, with “Years of education completed?”. Some respondents in CTN-0051 had high school information only listed in the baseline interview and the ASI. The demographic question used different coding schemes (e.g., high school graduate was coded as 12 in the ASI-Lite but the baseline survey coded it with the value 13). Education was harmonized into a categorical variable with education split into “Missing/Refused”, “Less than High School”, “High School/GED”, and “More than High School”. Missing ASI values for CTN-0051 were replaced with the self-reported demographic information for education. Instead of asking about current employment, however, ASI-Lite asked for “Usual employment status” which represents the majority of the last three years. Given that CTN-0051 also collected ASI-Lite, we harmonized these variables using only the ASI-Lite surveys and neglected the separate direct survey in CTN-0051 except for the education variable mentioned earlier. We obtained harmonized, derived variables to include Usual Employment Status.

Of note, CTN-0051 surveyed specifically whether someone was homeless through the PhenX Quality of Life survey. A similar item existed in the ASI-Lite, which reported whether or not someone had a stable living arrangement during the majority of the past three years. These items were moderately correlated in CTN-0051 (*φ* = 0.28). We harmonized the ASI-Lite living arrangements question (i.e., F4) into “No stable arrangement” or “Stable” (e.g., with a sexual partner, parents, friends, alone, etc.).

Marital status responses were categorized as “Separated/Divorced/Widowed”, “Married or Partnered”, “Single”, “Not Asked” or “Missing”.

The ASI-Lite also contained other questions in the domains of medical status, employment/support, alcohol/drugs, legal status, family/social relationships, and psychiatric status. These features were harmonized and are reported in domain-specific tables.

### Timeline Follow-Back (TLFB)

Self-reported drug and alcohol use histories were gathered using a standard method called Timeline Follow-Back (TLFB) [47], which involved systematically asking individuals whether and how they used a particular substance. Within the standard TLFB rubric, the CRFs were variable between studies. All studies used some combination of free text and structured questionnaires. The free text allowed for simple typos and misspellings (e.g., five spellings of ecstasy), multiple drugs as a single entry (e.g., “Vicodin/Percocet” or “Percocets and Vicodin”), entry of superfluous information such as units/amounts (e.g., “adderall 5 mg” or “adderall—2 tabs”), as well as complex text strings. While all studies asked about “any” alcohol use, only CTN-0051 specifically asked about the number of standard drinks. CTN-0027 asked about alcohol quantity with free text responses. This resulted in more than 1,100 distinct text strings describing daily alcohol use. The estimates of standard drinks necessitated hand curation. Alcohol consumption ranged from values corresponding to 0.5 drinks in a day (e.g., “1/2 12oz beer”, “1/2 beer”, “1/2 beer for birthday”, “1/2 of a beer”) and extended out to 49 standard drinks (i.e., “6pk beer & 1/2 gal. rum” in a day). All entries were converted to standard drinks using information provided by the National Institute on Alcohol Abuse and Alcoholism and Wikipedia. Bartending references were used to estimate the number of shots contained in larger containers. Ambiguous entries like “many glasses of wine” were coded as five standard drinks. CTN-0030 did not ask about the quantity of alcohol consumed. In CTN-0027, eight people were marked as having a drinking event but without information about the amount. Of these, four did not have a consistent pattern of heavy or light drinking, one was a consistently heavy drinker, and three were consistently light drinkers, so their values were imputed to match their normal drinking pattern.

Different substances were surveyed via structured questionnaires across the three studies. The same substances were surveyed in different ways in different studies: CTN-0027 had a nonspecific “opiates” entry in TLFB, but CTN-0030 and CTN-0051 had separate categories for heroin and prescription opioids. While all studies assessed substance use in classes other than opioids, as seen in Table 1, there were notable differences in the structured questions asking about daily drug use. CTN-0027 and CTN-0051 collected free text data on all “other” drugs. This resulted in a long list of other substances. CTN-0030 only gathered information on “other opioids”.

Two harmonized tables were built to hold TLFB data. One table, named *all_drugs*, contains an indicator of the method by which a drug was identified (i.e., either TLFB or UDS) for all the non-prescribed drugs. *all_drugs* contains information on the drug names after fixing typos and misspellings, parsing the cases where multiple drugs were entered as a single record, and removing superfluous text. The other table, named *tlfb*, collapses similar drugs into a “Drug Category” Table 1. For example, if an individual reported use of both crack cocaine and powder cocaine on a day, they would receive a single cocaine entry for a particular date of TLFB self-report. This method allows proper comparison with other studies that only surveyed the use of any cocaine without differentiating between the two types. This method also enables the creation of conceptually equivalent derived variables when there are different patterns of missing values. For calculating heroin vs. prescription opioids in CTN-0027, we used a method derived from existing literature [48] that inferred that “opiates” in the CRF were conceptually identical to heroin, and oxycodone was conceptually identical to prescription opioids. This method of deriving conceptually equivalent derived variables has been commonly used in CDM literature [18], where standard vocabularies have equivalent classes and nested derived classes. While there is a lack of community-wide standardization in this field, our attempt represents a proposal that would standardize similar concepts in TLFB reports in all SUD treatment clinical trials.

Additional complications in TLFB data involved stipulated route of use, which also was collected in different ways. In CTN-0027 and CTN-0030, route and quantity were surveyed as a mixture of structured data and free texts, and in CTN-0051, route of use was categorical (i.e., 1-oral, 2-nasal, etc.) for every day of the survey. Given the heterogeneity of the data, we did not attempt to harmonize the route and quantity of use between the three studies using TLFB.

### Urine Drug Screening (UDS)

UDS is a relatively uniform assay across these trials. A typical 10-drug UDS test was conducted as an enzyme-linked immunosorbent assay in all three studies, which targeted Amphetamines, Benzodiazepines, Methadone, Oxycodone, Cocaine, Methamphetamine, Opiates (at a threshold of 300 or 2000ng/mL), Cannabinoids, Propoxyphene, and Buprenorphine. These assays also often included the temperature of the sample to ensure the sample was produced at the time of collection and whether the urine collection was supervised. In CTN-0051, there were flags indicating clinicians’ suspicions on whether the sample produced was reliable, and if any concerns came up, a second sample was collected. In CTN-0027 and CTN-0030, only one sample was collected and recorded per visit. In addition, CTN-0051 tested for two substances (i.e., Barbiturate and MDMA) that were unavailable in the other two studies. As with TLFB, we produced two harmonized datasets for the UDS. The aforementioned *all_drugs* table kept the original drug labels. The other table, named *uds*, grouped drugs of the same category (see Table 1). Opioid tests of different thresholds (300 or 2000) were harmonized into a concept of “any positive UDS for morphine metabolite.” Because urine temperatures in range were collected in all three studies, they were harmonized into a “Temperature in Range” concept, which is reported in a table named *uds_temp*.

Another interesting feature of UDS was that the immunosorbent test result was not always apparent at the time of testing, yielding “unclear” (CTN-0027: N = 7, CTN-0030: N = 6) or “invalid” (CTN-0051: N = 3) entries. A small number of entries were “not assessed.” These tests were for individual substances that were missing when the other substances were assessed (CTN-0027: N = 13, CTN-0030: N = 1), presumably also because the immunosorbent test did not yield a readable result only for a particular substance. All of these circumstances were treated as a negative UDS result.

There were several other minor discrepancies between the three studies. Baseline UDS was collected between the initial screen date and randomization date in CTN-0027 and CTN-0030, but in CTN-0051, it was collected after initiation of detoxification. This tended to bias the number of opioid-positive screens in the CTN-0051 sample to negative. Buprenorphine records in UDS did not exist in CTN-0027, and in CTN-0030, they were only scheduled in phase 1 at weeks 10, 12, and at the final visit, and in phase 2, at weeks 22 and 24. Because the “final visit” data for phase 1 of CTN-0030 could be triggered by treatment failure, which could happen at any point, Buprenorphine records were scattered throughout the early weeks of CTN-0030.

There were notable issues in the UDS data, which required extremely time-intensive hand curation. The most problematic was the presence of multiple UDSs on the same date. While a few entries had notes justifying the duplicates, the vast majority did not. Often, the discrepancies appeared to be typos in the date, which could be resolved by looking at the time stamps when the UDSs were logged. In other cases, records seemed to be intentionally duplicated to create “end of study” records when a person left before the end of the trial.

### Nicotine use

All studies used the Fagerstrom Test for Nicotine Dependence score [49] to assess the severity of nicotine dependence. The details on the coding of the FTND were missing from the primary documentation for CTN-0027. However, subject matter experts on the CTN-0094 team were able to recreate the coding for the questions.

### Withdrawal

CTN-0027 and CTN-0030 used the Clinical Opiate Withdrawal Scale (COWS), which contained 11 questions and is scored into four categories: mild, moderate, moderately severe, and severe. CTN-0051 used the Subjective Opioid Withdrawal Scale (SOWS), which has 16 items and is scored into three categories: mild, moderate, and severe. We harmonized both scales into a new Opioid Withdrawal Severity concept that had four categories which were assigned using rules (in this order): severe (COWS ≥ 25, SOWS ≥ 21), moderate (COWS ≥ 13, SOWS ≥11), mild (COWS ≥5, SOWS ≥ 1), and none (COWS ≥ 0, SOWS = 0), with moderately severe and severe counted as the same category in COWS. Participants were repeatedly assessed for withdrawal symptoms. Two harmonized tables are provided in the public.ctn0094data R package. One, named *withdrawal*, has the complete set of withdrawal measures. The other, which is redundant but useful for analysts, named *withdrawal_pre_post*, has the closest measurements before and after initial treatment induction.

### Medical and psychiatric history

Medical histories in all three studies were surveyed using a routine medical history and physical exam. All three studies surveyed psychiatric and SUD history but they used combinations of different instruments. All studies asked the “serious depression”, “serious anxiety/tension” and “experienced hallucinations” not caused by drug use questions from the ASI-Lite. Given the modest agreement between the medical history report of psychiatric conditions and the ASI (e.g., the *φ* statistic for the two metrics of schizophrenia is 0.27), both sources of information were separately harmonized. CTN-0027 and CTN-0030 used a DSM-IV Checklist and CIDI, and CTN-0051 used a DSM-5 Checklist and an updated version of CIDI. The exact conditions surveyed were slightly different: for example, CTN-0030 had an entry for “psychotic disorder” but no schizophrenia, only CTN-0030 surveyed PTSD, CTN-0027 did not assess ADHD, and all three studies allowed free text entries for other psychiatric and medical conditions. These “semi-structured” text fields present concerns because without a measurement scale, it is unclear where values represent a symptom or a clinically relevant disorder. CTN-0051 had explicit documentation of a history of suicidal behavior, but CTN-0027 and CTN-0030 only documented suicidality if it was significant enough to make study participation inappropriate. None of the studies had explicit documentation of previous drug overdose history. Details of previous treatment attempts (number, method, frequency, duration, and short-term outcomes) were not available. Given the relative heterogeneity, we derived the following binary harmonized categories for past medical and psychiatric diagnoses that exist in all three studies and are conceptually equivalent: schizophrenia or other psychotic disorder, major depressive disorder, bipolar disorder, anxiety disorder, epilepsy, and other clinically significant neurological damage/disorder.

Baseline diagnoses of SUD were also heterogeneous. DSM-IV criteria and categories were used for CTN-0027 and CTN-0030, which contained checks for either substance abuse or dependence. In CTN-0051, DSM-5 was used and the diagnoses were marked as binary (“Substance Use Disorder”). We harmonized these binary categories into a single binary flag for either a diagnosis of DSM-IV substance abuse or dependence or positive for a DSM-5 use disorder (“Diagnosis of Substance Use Disorder”).

### Quality of life measures

CTN-0027 and CTN-0030 used the SF-36 [50, 51] which covered eight health concepts: physical functioning, bodily pain, role limitations due to physical health problems, role limitations due to personal or emotional problems, emotional wellbeing, social functioning, energy/fatigue, and general health perceptions as well as a single item that provided an overall perceived change in health. This instrument is used widely for calculating important health systems metrics such as Quality Adjusted Life Years (QALY) using a derived preference measure called SF-6D [52]. CTN-0051 used the EuroQoL to assess five domains: Mobility, Self Care, Usual Activities, Pain/Discomfort, and Anxiety/Depression. Each of which is assessed using three possible responses. Given the considerable difficulty in the interconversion of these two measures in summary domains, we did not derive common concept variables except for the severity of chronic pain at baseline. In the past, the presence or absence of pain was considered a predictor of treatment response [53]. Here, the six levels of pain assessed by the SF-36 questions “How much bodily pain have you had during the past 4 weeks?” were grouped into three levels approximating the responses in the EuroQoL. The SF-36 response of “None” was considered “No Pain”; “Very Mild”, “Mild”, and “Moderate” were grouped as “Very Mild to Moderate Pain”; “Severe” and “Very Severe” were grouped as “Severe Pain”. The EuroQoL responses “I have no pain or discomfort”, “I have moderate pain or discomfort”, and “I have extreme pain or discomfort” were mapped on the same labels as the SF-36. Of note, both CTN-0030 and CTN-0051 excluded people who required ongoing pain management that required opioids. CTN-0030 further excluded people who had experienced a major pain event within the past 6 months or were prescribed methadone >40 mg a day for pain.

### Survey of risky behaviors

All three studies surveyed risky behavioral patterns at baseline. CTN-0027 and CTN-0030 used the Risk Behavior Survey (RBS) [54], which assessed behavior surveyed on a continuous scale of days of use for cocaine, heroin, and other substances used in the past 30 days. Furthermore, the RBS detailed how many days each substance was injected. It also surveyed same-sex behavior and unprotected sex with substantial detail. CTN-0051 used a different survey called the Risk Assessment Battery (RAB) [55], initially used in HIV vaccine trials to assess risk of HIV transmission. This survey also assessed the number of days an individual used various substances of abuse, but rather than having a continuous number of days, categories were used. To harmonize these two datasets, we converted the categorical assessments into an estimated number of days of use in a 30-day period using a rule specifying that every day meant 30 days, a few times each week as 15 days, a few times as 5 days, and not at all as 0 days. We also created harmonized data variables indicating the maximum number of days of use across all intravenous (IV) drugs, the maximum number of drug-use events, the total amount of drug exposure for the most used IV drug, and many indicators of sexual risk behaviors, including the number of sex partners, unprotected sex, and the number of same-sex partners. A small number of inconsistencies were noted in the sex behavior data. For example, for three people, the total number of male partners plus the total number of female partners did not equal the total partners. Given that we have no third source of “truth,” these inconsistencies were left in the data reported in the public.ctn0094data package. We omitted questions specific to HIV tests as they were not assessed in either survey.

### Randomization

CTN-0027 and CTN-0051 used a parallel-group design with one randomization for all participants. In CTN-0030, two randomization events occurred, each with an associated randomization date. Because of this, in the public.ctn0094data package, each person has two randomization records. The two randomization dates for CTN-0027 and CTN-0051 are identical. Each randomization record includes an indicator for the date and the treatment assignment (i.e., “Inpatient BUP”, “Inpatient NR-NTX”, “Methadone”, “Outpatient BUP”, “Outpatient BUP + Enhanced Medical Management”, “Outpatient BUP + Standard Medical Management”).

### Treatment and medication doses

The treatment assignment can be derived from the randomization table. To assist analysts, a table named *meta_study_length* was created that includes a timeline for each trial. It includes the treatment group along with indicators for the study phase (to support CTN-0030 data analysis), study stage (e.g., stabilization, taper, or post-medication follow-up), and duration in weeks.

The three studies had heterogeneous ways of recording medication doses, reflecting the peculiarities of the treatment delivery system. Some CTN-0027 participants recorded prescription doses weekly, while others logged the daily doses, likely reflecting that the participants were daily participants in federally licensed treatment programs. For some participants, multiple logs existed on the same day, especially during induction, likely reflecting in-clinic and take-home/add-on doses. We harmonized the dose logs from the source data into a form where each day has a prescribed dose associated. We then produced harmonized derived data containing the total treatment drug in mg or the value 1 for injection treatments in CTN-0051. A total of 100 cases were observed where the data indicated a patient received a single dose of the “other” drug in the midst of being treated with the drug assigned at randomization. In all cases, the dose corresponded to the treatment drug. Therefore, these data entries were corrected to reflect the drug assigned at randomization.

### Dates

One of the major challenges involved in appropriately harmonizing clinical trial data involves deriving conceptually equivalent dates. While clinical trial design-specific events (i.e., dates of consent, screening, randomization, first follow-up visit, etc.) appear at first glance to be the same for all clinical trials, in reality, they often represent conceptually different times in terms of treatment delivery.

**Fig 5:**
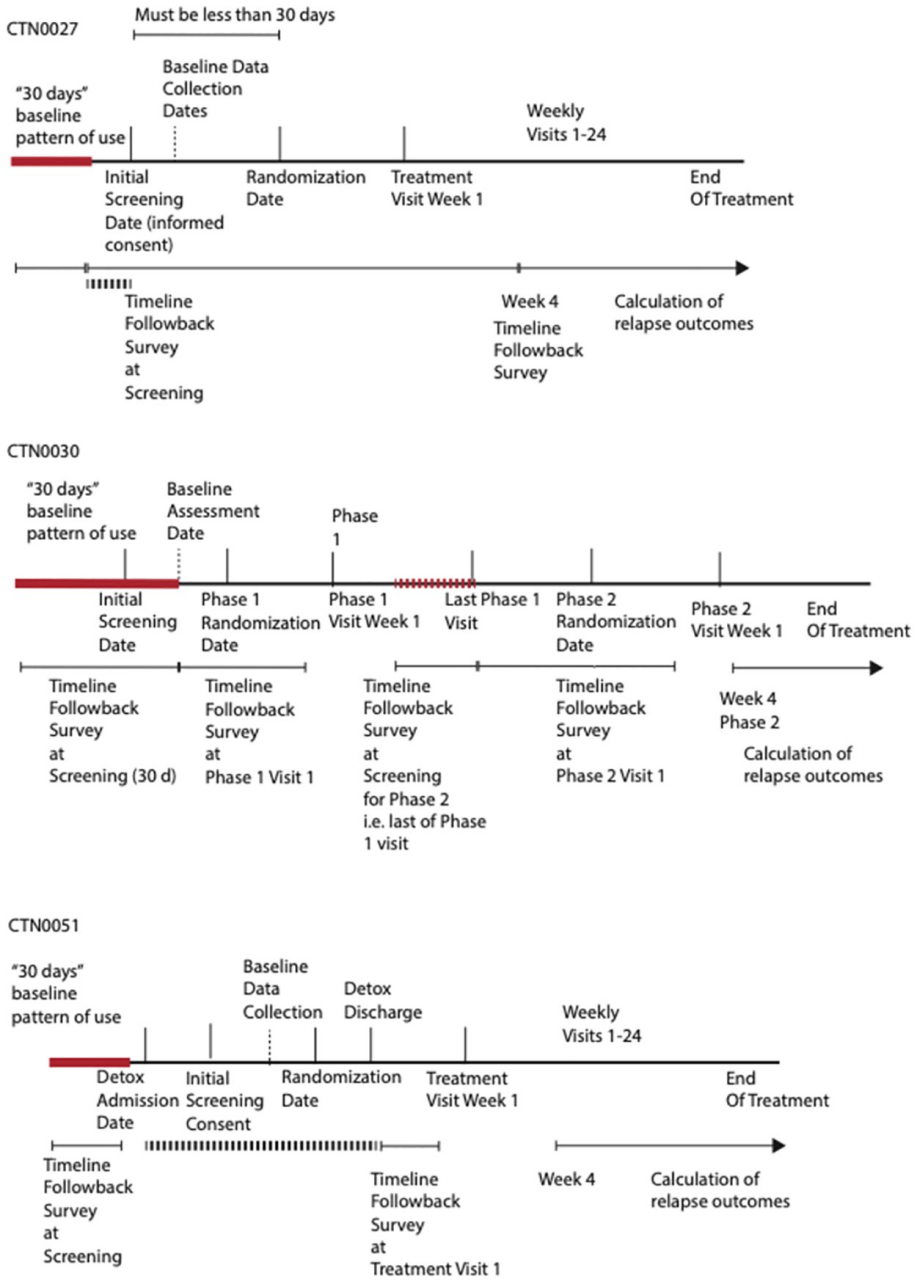
Dates and designs of studies.

In particular, the definitions of what constitutes a “baseline” survey of a patient’s drug use patterns before treatment engagement differed between the three studies (Fig 5). In CTN-0027, TLFB forms had dates to which each entry refers. There was, therefore, a natural order to TLFB measures uncoupled from the dates when the forms themselves were filled out (Assessment Date). When a patient entered into treatment, the study team first explained and consented the patient (Initial Screening Date), then a series of evaluations occurred (Secondary Screening Visits) where various baseline measures were taken. Once all inclusion criteria were met, patients were randomized (Randomization Date). There was a natural gap between the end of the first TLFB form and the Initial Screening Date because TLFB was made at a later Secondary Screening Visit that referred to the patient’s drug use prior to treatment engagement (i.e., “in the 30 days before you started treatment”, solid red line in Fig 5). This gap was then filled during a subsequent survey in Week 4, which backfilled all entries since the last survey.

In CTN-0030, the patients were screened first on the Initial Screening Date, and only if they met certain screening criteria (i.e., predominantly using prescription opioids as opposed to heroin), they later had a thorough baseline assessment on the *Baseline Assessment Date*. On that date, 30 days of prior TLFB was collected. If the patients met the criteria for entry, they were randomized into Phase 1 on the *Randomization Date*. At some point during Phase 1, they were deemed eligible to enter Phase 2, and between the last Phase 1 and the first Phase 2 visit, would be the *Phase 2 Randomization Date*. If we only considered the Phase 2 trajectory, there was a debate as to whether the baseline referred to Phase 1 baseline (solid red line) or a new baseline when the patient failed Phase 1 treatment (Phase 2 baseline, dotted red line). Considering that the Phase 2 baseline might be influenced by the treatment given in Phase 1, in the initial harmonization, we defined Phase 1 baseline as the baseline survey period.

In CTN-0051, the patients entered inpatient detoxification facilities on their own on the Detox Admission Date, and the team started engagement with the patients after they started the detoxification procedure (Initial Screening Date), followed by a series of patient engagements during that period until patients were deemed eligible for the study and randomized (Randomization Date). On either the Initial Screening Date or a Secondary Screening Visit Date, a TLFB form was filled out. Logically, the study team wanted to survey drug use patterns prior to admission into the detox facility (solid red line). Furthermore, once the patients were admitted into the detox facility, their self-reports would presumably be negative; therefore, a large portion of the longitudinal drug use survey was missing (dotted black line). Finally, after the patient was discharged from the detox facility and inducted onto either XR-NTX or BUP, their use pattern was back-filled up to the point of detox discharge at Treatment Follow-up Visit Week 1. In particular, because the study was pragmatic and the study team did not dictate how long the detox hospitalization length of stay should be, the backfilling prior to Treatment Visit 1 was of varying lengths.

The uniform concept here of “baseline” was the initial survey of 30 days prior to any treatment engagement, but this was difficult to calculate, as this period referred to none of the universal clinical trial design dates (i.e., Initial Screening Visit Date, Randomization Date, etc.). We therefore harmonized the baseline period in all three studies to a new concept (red dotted lines) called End of Baseline Survey, determined by the date orders of the forms, that is, the first batch of 30 days of TLFB collected prior to any study engagement. There was usually a gap between this period and the Randomization Date, where treatment was selected at random. We harmonized the lengths of this period into a concept called “Number of Days from Baseline Survey to Randomization,” as we felt that this might have interesting clinical implications, such that the delay, for whatever reason, between the initial engagement in treatment and treatment selection could be predictive of treatment success.

Similar to the definition of “baseline,” the definition of the start of the treatment and the order of the weeks was also not clearly defined at first glance. In all three studies, after randomization, in theory, patients would be followed once a week, but in practice, the visits often had long gaps between them. The study teams might also refer to one visit as Week 1, even though it occurred more than seven days after the Randomization Date. Given that Randomization Date had a clear clinical interpretation (the day on which a treatment assignment decision was made), we decided to discard orders of follow-up dates given in the original investigators’ dataset and instead use calendar days as a reference: that is, 0–6 days after Randomization Date was Week 1 in all three studies, 7–13 days was Week 2, etc. This method also had the added advantage that it could be applied to any number of longitudinal studies with varying frequencies of stipulated follow-up visits rather than relying on a prespecified schedule. If multiple visits occurred within the same week, they would both be used in any of the derived variables pertinent to that week. For example, a “use week” is defined, following the CTN-0051 convention, as having any positive nonprescribed opioid urine toxicology data in any of the visits during a particular week. A “relapse” is defined as four consecutive use weeks on or after Week 4.

Finally, a large number, though a relatively small proportion, of dates was incorrectly entered (N > 550 records across all three studies). Some were very clearly incorrect, for example, where the survey date was a year or more off from the first date of the TLFB surveyed at baseline (i.e., 3/1/2009 was mistyped as 3/1/2007). These were corrected to reflect the most likely date. A small number of individuals (N = 7) had the survey date and the last day of the survey misaligned by one day (i.e., survey date 3/1/2009, but TLFB date was 3/2/2009). Survey dates were moved to the last day of TLFB report in these cases. Finally, there were overlaps in TLFB report dates surveying the exact dates multiple times at multiple visits (e.g., at baseline and Week 1), etc. We defined “positive” as any positive report on a particular day.

## Results

### Social and demographic characteristics

#### Age

**Fig 6:**
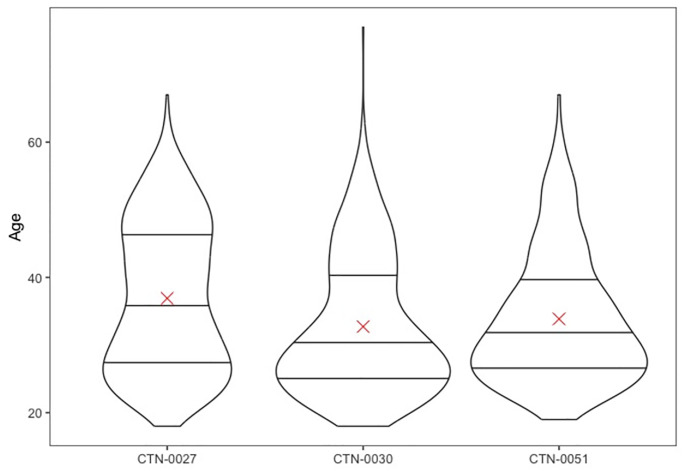
Violin plot of age. At Screening for 2,492 People Seeking Care for Opioid Use Disorder by Trial (CTN-0027 N = 1,269, CTN-0030 N = 653, CTN-0051 N = 570). Reference lines are at the 25th, 50th, and 75th Percentile and an X at the Mean.

Small but statistically significant (p<0.001) differences were noted between the ages of participants across the three trials. The typical age in CTN-0027 (mean = 37) was higher than in either CTN-0030 (mean = 33; p<0.001) or CTN-0051 (mean = 34; p<0.001). This effect was driven by a bimodal age distribution in CTN-0027 (see Fig 6), whereby the younger bolus of subjects had a similar age distribution across all studies, but CTN-0027 has a second population centered around age 50.

#### Sex

Statistically significant differences (p<0.001) were noted in the proportion of female participants across the three trials. The proportion of females in CTN-0030 (40%) was higher than in either CTN-0027 (32%, p = 0.002) or CTN-0051 (30%, p<0.001). However, the differences between CTN-0027 and CTN-0051 were not noteworthy (p = 0.310).

#### Race and ethnicity

As can be seen in Table 2, there are notable differences in the frequencies of the race (p<0.001) and ethnic (p<0.001) groups. These statistically significant differences in frequency are driven by many factors, including a relatively small proportion of Whites and many people in the “other” group in CTN-0027, an overabundance of Whites in CTN-0030 and a relatively large group of Blacks in CTN-0051.

**Table 2:** Race and ethnicity by CTN trial number.

	CTN-0027 (N = 1269)	CTN-0030 (N = 653)	CTN-0051 (N = 570)	Overall (N = 2492)
**Race**				
Refused/Missing	6 (0.5%)	1 (0.2%)	6 (1.1%)	13 (0.5%)
Black	128 (10.1%)	21 (3.2%)	73 (12.8%)	222 (8.9%)
Other	229 (18.0%)	35 (5.4%)	70 (12.3%)	334 (13.4%)
White	906 (71.4%)	596 (91.3%)	421 (73.9%)	1923 (77.2%)
**Ethnicity**				
Hispanic	206 (16.2%)	31 (4.7%)	99 (17.4%)	336 (13.5%)
Not Hispanic	1063 (83.8%)	622 (95.3%)	471 (82.6%)	2156 (86.5%)

#### Education, employment, and relationship status

Only a small subset of CTN-0027 participants (13%) completed the ASI-Lite. Table 3 summarizes the differences in demographic features which were observed between the studies: education (p = 0.002), employment (p<0.001 using Fisher’s exact test with Monte Carlo methods), living arrangements (p<0.001), and relationship status (p<0.001). Overall, a large plurality of individuals who entered treatment were employed, either part time or full time, with a higher proportion being employed in CTN-0030, and only a small number of individuals having a history of unstable living conditions. CTN-0030 also had a sample with a larger proportion of individuals who were married or partnered compared to the other two studies. Interestingly, two demographic features, education (N = 623) and marital status (N = 613), were asked on the medical intake as well as the ASI. The agreement on the two sources was 88% for education and 93% for marital status.

**Table 3:** Education, employment, living arrangements and relationship status by trial.

	CTN-0027 (N = 162)	CTN-0030 (N = 651)	CTN-0051 (N = 569)	Overall (N = 1382)
Education				
HS/GED	59 (36.4%)	252 (38.7%)	227 (39.9%)	538 (38.9%)
Less than HS	44 (27.2%)	101 (15.5%)	123 (21.6%)	268 (19.4%)
More than HS	59 (36.4%)	298 (45.8%)	219 (38.5%)	576 (41.7%)
Employment				
Full Time	68 (42.0%)	410 (63.0%)	268 (47.1%)	746 (54.0%)
Other	7 (4.3%)	16 (2.5%)	24 (4.2%)	47 (3.4%)
Part Time	34 (21.0%)	115 (17.7%)	116 (20.4%)	265 (19.2%)
Student	1 (0.6%)	28 (4.3%)	10 (1.8%)	39 (2.8%)
Unemployed	52 (32.1%)	82 (12.6%)	151 (26.5%)	285 (20.6%)
Usual Living Arrangements				
Stable	153 (94.4%)	649 (99.7%)	533 (93.7%)	1335 (96.6%)
Not Stable	9 (5.6%)	2 (0.3%)	36 (6.3%)	47 (3.4%)
Relationship Status				
Married or Partnered	26 (16.0%)	186 (28.6%)	55 (9.7%)	267 (19.3%)
Never married	88 (54.3%)	326 (50.1%)	391 (68.7%)	805 (58.2%)
Separated/Divorced/Widowed	48 (29.6%)	139 (21.4%)	123 (21.6%)	310 (22.4%)

This table includes the subset of people who have known education, employment, living arrangements, and relationship status data.

### Timeline follow-back

Harmonizing all free text entries of self-reported TLFB yielded 32 different classes of substances, showing a pattern of self-reported use of both prescribed (antidepressant, antihistamine) and non-prescribed substances (Table 4). While many of the same drugs were reported in all three studies, several differences are noteworthy. First, the free-text entry in CTN-0027 encouraged self-reports of prescribed medications such as antidepressants and antibiotics, though these are relatively rare. Because CTN-0030 did not allow for the free text entry of all other drugs, only other opiates, there are no records of muscle relaxants, hallucinogens, and/or MDMA. Inhalants, K2/Spice, and Kratom first appeared in the more recent CTN-0051 data, though they are relatively rare. In CTN-0051, methamphetamine use was combined with amphetamine use, whereas they are different categories in the other two studies. In the structured TLFB questions, CTN-0051 asked about “Hallucinogens, including MDMA/ecstasy”. The free-text drug list in CTN-0027 allowed for the identification of unspecified hallucinogens separately from MDMA. Given that the large number of substances listed in Table 4 makes the data difficult to work with, we harmonized the raw drug use data in the three studies to standardized derived concepts. The patterns in the harmonized data are shown in Table 5.

**Table 4:** Timeline follow-back by trial—Ungrouped.

	CTN-0027	CTN-0030	CTN-0051
Acetaminophen	24	3	1
Alcohol—Any Drinking	0	4902	0
Alcohol—Heavy Drinking	2112	0	4151
Alcohol—Light Drinking	6197	0	2422
Amphetamine	610	142	3446
Antibiotic	13	0	0
Antidepressant	16	0	0
Antiemetic	7	1	0
Antihistamine	11	0	2
Antipsychotic	69	0	1
Barbiturate	1	0	8
Benadryl	1	0	0
Benzodiazepine	3327	3124	3526
Buprenorphine	23	99	2186
Caffeine	0	0	1
Cathinones	1	0	6
Clonidine	72	0	0
Cocaine	5494	935	3924
Codeine	27	490	4
Crack	10628	0	3029
Dextromethorphan	0	31	1
Fentanyl	7	392	2
Gabapentin	26	0	6
GHB	0	0	1
Hallucinogen	16	0	189
Heroin	49753	368	30857
Hydrocodone	58	12307	0
Hydromorphone	10	490	0
Inhalant	0	0	4
K2	0	0	355
Kratom	0	0	21
MDMA	16	0	189
Meperidine	0	33	0
Methadone	2539	2684	518
Methamphetamine	2919	263	1
Methylphenidate	11	0	0
Morphine	50	940	0
Muscle Relaxant	169	0	12
Nalbuphine	0	11	0
Opioids (Not Specified or Other Opioids)	26	2	4660
Opium	0	368	0
Oxycodone	4697	16369	0
Oxymorphone	0	11	0
PCP	20	0	10
Propoxyphene	43	198	0
Pseudoephedrine	2	0	0
Sedative-Hypnotic	18	351	263
Suboxone	276	6126	0
THC	18866	10495	17064
Tramadol	14	646	0
Trazodone	22	0	0
Trycyclic-Antidepressant	14	0	0
Unknown	2	2	0

**Table 5:** Timeline follow-back by trial—Drug names grouped.

	CTN-0027	CTN-0030	CTN-0051
Alcohol	8309	4902	6573
Amphetamine	3513	399	3447
Analgesic	50	3	7
Antibiotic	13	0	0
Antidepressant	38	0	0
Antiemetic	7	1	0
Antihistamine	11	0	2
Antipsychotic	69	0	1
Benadryl	1	0	0
Benzodiazepine	3327	3124	3526
Buprenorphine	132	382	1236
Caffeine	0	0	1
Cathinones	1	0	6
Clonidine	72	0	0
Cocaine	16120	935	6699
Dextromethorphan	0	31	1
GHB	0	0	1
Hallucinogen	16	0	189
Heroin	49753	368	30857
Inhalant	0	0	4
K2	0	0	355
Kratom	0	0	21
MDMA/Hallucinogen	16	0	189
Methadone	2426	2684	518
Methylphenidate	11	0	0
Muscle Relaxant	169	0	12
Opioid	4922	28982	4666
PCP	20	0	10
Pseudoephedrine	2	0	0
Sedatives	19	351	271
THC	18866	10495	17064
Unknown	2	2	0

Several noteworthy patterns emerged. CTN-0030, in particular, had a sample that consisted primarily of individuals who used prescription opioids, though about 27.6% of the individuals reported at least one dose of methadone at some point. In both CTN-0027 and CTN-0051, most positive individual self-reports were of heroin use. Of note, about 60% of cocaine users in both CTN-0027 and CTN-0051 were using crack cocaine, a distinction that was not surveyed in CTN-0030. Use of sedative-hypnotics and hallucinogens was relatively rare in all three studies.

Finally, when exploring the days of use, rather than drug use events, other than the aforementioned difference between high frequency opioid use (CTN-0030) vs. heroin use (CTN-0027 and CTN-0051), the overall patterns of use were not substantially different between the three cohorts. Because drug use patterns may vary across the days of the week (e.g., some people may binge on Friday and/or Saturday nights), the count of days of use, shown in Table 6, reflects totals that are done out of 28 consecutive days (4 weeks times 7 days is a 28 day-time window) rather than the 30 day month of observation done in other types of studies. Of note, a small number of individuals used cocaine or amphetamine derivatives, but the overall median number of days of use across the three studies was zero, with a median of three days of use among the cocaine users and a median of two among the amphetamine users, meaning that a pattern of regular use of stimulants was unusual in all three studies. In general, patients used either heroin or opioids daily, with a median number of days of 27 days in both CTN-0027 and CTN-0030, and 18 days in CTN = 0030. However, a substantial minority of individuals had sporadic opioid-free days: one standard deviation is six days of opioid use in both CTN-0027 and CTN-0030, and seven days of opioid use in CTN-0051.

**Table 6:** Timeline follow-back by trial summarizing days of use, out of 28 days, for people who used each substance.

	CTN-0027			CTN-0030			CTN-0051		
	Q1	Median	Q3	Q1	Median	Q3	Q1	Median	Q3
Alcohol	1	2	6	1	2	5	1	3	9
Amphetamine	1	2	5	1	1	3	1	3	6
Benzodiazepine	1	2	3	1	2	6	1	3	8
Cocaine	1	3	10	1	1	2	2	3	7
Hallucinogen	1	1	1	NA	NA	NA	1	1	3
Heroin	23	27	28	1	2	2	12	19	23
MDMA/Hallucinogen	2	2	3	NA	NA	NA	1	1	3
Opioid	2	9	24	24	27	28	2	6	14
Sedatives	1	2	2	1	1	6	2	3	7
THC	1	3	18	2	6	22	1	6	17

### Urine drug screening

Patterns of harmonized UDS data were tabulated in Tables 7 and 8. The median number of UDS a participant provided throughout all three studies is 14. Counting follow-up and buprenorphine taper periods, the three studies had approximately the same duration of follow-up. However, there were fewer UDS records in CTN-0030. In phase 2 of CTN-0030, there were relatively more records, reflecting that most of the participants were not able to sustain abstinence when buprenorphine was tapered. We also examined the patterns of individual substances of abuse in harmonized UDS records. A small number of UDS reports (≅ .06% for CTN-0027 and CTN-0030 and ≅ .05% for CTN-0051) were missing or unclear for at least one substance, even though the patients attended the follow-up visits and had provided UDS for other substances. Approximately 5% of the UDS were positive for amphetamines; 17% were positive for benzodiazepines, with a higher prevalence in CTN-0030; buprenorphine was regularly detected (51.2%) in CTN-0051; methadone was frequently detected (32%) with the effect being driven by the majority of records in CTN-0027 (50.4%); oxycodone was frequently detected (10%) with most reports coming from CTN-0030 (39.9%); cocaine was detected in 23% of UDSs, with a higher prevalence in CTN-0027 (31.5%); 5% of records detected methamphetamine; and cannabis use was prevalent in all three studies at about 25%. Finally, urine of the wrong temperature (i.e., doctored urine) was a very rare occurrence, happening 0.23% of the time in any of the three studies (Table 9).

**Table 7:** Number of urine drug screening results by trial.

	CTN-0027 (N = 1269)	CTN-0030 (N = 647)	CTN-0051 (N = 570)	Overall (N = 2486)
UDS Records Per Person				
Median (Q1—Q3)	21 (6.0–25)	7.0 (4.0–15)	14 (7.0–25)	14 (5.0–24)

**Table 8:** Number and percent of positive urine drug screening results by trial.

	CTN-0027		CTN-0030		CTN-0051		Total	
	Freq	Percent	Freq	Percent	Freq	Percent	Freq	Percent
Alcohol	148	0.7%	0	0.0%	0	0.0%	148	0%
Amphetamine	1150	5.5%	254	4.0%	310	3.6%	1714	5%
Barbiturate	0	0.0%	0	0.0%	227	2.6%	227	1%
Benzodiazepine	3089	14.7%	2049	32.6%	1116	12.9%	6254	17%
Buprenorphine	0	0.0%	374	5.9%	4420	51.2%	4794	13%
Cocaine	6595	31.5%	704	11.2%	800	9.3%	8099	23%
MDMA	0	0.0%	0	0.0%	20	0.2%	20	0%
Methadone	10558	50.4%	522	8.3%	429	5.0%	11509	32%
Methamphetamine	1462	7.0%	177	2.8%	308	3.6%	1947	5%
Opioid	8317	39.7%	1850	29.4%	1111	12.9%	11278	31%
Oxycodone	947	4.5%	2508	39.9%	151	1.7%	3606	10%
Propoxyphene	173	0.8%	164	2.6%	0	0.0%	337	1%
THC	4988	23.8%	2284	36.3%	1795	20.8%	9067	25%

**Table 9:** Temperature urine drug screening results by trial.

	CTN-0027 (N = 20961)	CTN-0030 (N = 6287)	CTN-0051 (N = 8638)	Overall (N = 35886)
Temperature In Range				
No	25 (0.1%)	16 (0.3%)	40 (0.5%)	81 (0.2%)
Yes	20732 (98.9%)	6242 (99.3%)	8598 (99.5%)	35572 (99.1%)
Missing	204 (1.0%)	29 (0.5%)	0 (0%)	233 (0.6%)

### Nicotine use

As can be seen in Table 10, most people (85%) in all three studies were smokers. On average, smokers had a score of 5.5 (median = 6, first quartile = 3, third quartile = 8) on the Fagerstrom Test for Nicotine Dependence, indicating between “low to moderate dependence” and “moderate dependence”. A small but statistically significant difference in the smoking rate was observed between the people in CTN-0030 versus the other trials (13%, p<0.001).

**Table 10:** Smoking details.

	27 (N = 1264)	30 (N = 653)	51 (N = 570)	Overall (N = 2487)
**Smoking Status**				
Smoker	1129 (89.3%)	489 (74.9%)	489 (85.8%)	2107 (84.7%)
Nonsmoker	135 (10.7%)	164 (25.1%)	81 (14.2%)	380 (15.3%)
**Cigarettes per Day**				
None	135 (10.7%)	164 (25.1%)	81 (14.2%)	380 (15.3%)
10 OR LESS	353 (27.9%)	116 (17.8%)	194 (34.0%)	663 (26.7%)
11–20	576 (45.6%)	228 (34.9%)	201 (35.3%)	1005 (40.4%)
21–30	153 (12.1%)	113 (17.3%)	81 (14.2%)	347 (14.0%)
31 OR MORE	47 (3.7%)	32 (4.9%)	13 (2.3%)	92 (3.7%)
**Fagerstrom Test for Nicotine Dependence**				
NA	135 (10.7%)	164 (25.1%)	81 (14.2%)	380 (15.3%)
0	61 (4.8%)	40 (6.1%)	35 (6.1%)	136 (5.5%)
1	76 (6.0%)	44 (6.7%)	31 (5.4%)	151 (6.1%)
2	101 (8.0%)	50 (7.7%)	39 (6.8%)	190 (7.6%)
3	154 (12.2%)	55 (8.4%)	66 (11.6%)	275 (11.1%)
4	192 (15.2%)	49 (7.5%)	77 (13.5%)	318 (12.8%)
5	188 (14.9%)	67 (10.3%)	87 (15.3%)	342 (13.8%)
6	155 (12.3%)	72 (11.0%)	57 (10.0%)	284 (11.4%)
7	112 (8.9%)	65 (10.0%)	46 (8.1%)	223 (9.0%)
8	63 (5.0%)	35 (5.4%)	36 (6.3%)	134 (5.4%)
9	19 (1.5%)	9 (1.4%)	10 (1.8%)	38 (1.5%)
10	8 (0.6%)	3 (0.5%)	3 (0.5%)	14 (0.6%)
Missing	0 (0%)	0 (0%)	2 (0.4%)	2 (0.1%)

### Withdrawal symptoms

All three studies obtained records of withdrawal symptoms before and after induction, and several times after the first month of treatment (Table 11). We tabulated withdrawal symptoms before and after induction (i.e., the first dose of study medication). Most of the participants entering the studies reported at least mild to moderate levels of withdrawal prior to entry into the study. Also of note is that 29.3% of CTN-0051 patients reported severe withdrawal symptoms pre-induction, whereas severe withdrawal was a relatively rare occurrence in the other two studies. Post-induction, most CTN-0030 patients in Phase 2 reported no withdrawal symptoms (80.9%). A plurality of patients reported mild withdrawal (50.4%) in CTN-0027. The patterns of the description of withdrawal symptoms had a wider range in CTN-0051, with 9.7% of patients reporting severe withdrawal after induction, in particular. Within the severe group, XR-NTX assignment was 22% and in the moderate group after induction, XR-NTX assignment was 25%.

**Table 11:** Withdrawal symptoms around the time of initial treatment by trial.

	CTN-0027		CTN-0030		CTN-0051		Overall	
	Pre (N = 1268)	Post (N = 1150)	Pre (N = 653)	Post (N = 613)	Pre (N = 570)	Post (N = 462)	Pre (N = 2491)	Post (N = 2225)
Severity								
None	21 (1.7%)	521 (45.3%)	2 (0.3%)	496 (80.9%)	20 (3.5%)	62 (13.4%)	43 (1.7%)	1079 (48.5%)
Mild	672 (53.0%)	547 (47.6%)	349 (53.4%)	110 (17.9%)	239 (41.9%)	298 (64.5%)	1260 (50.6%)	955 (42.9%)
Moderate	553 (43.6%)	79 (6.9%)	295 (45.2%)	7 (1.1%)	144 (25.3%)	57 (12.3%)	992 (39.8%)	143 (6.4%)
Severe	22 (1.7%)	3 (0.3%)	7 (1.1%)	0 (0%)	167 (29.3%)	45 (9.7%)	196 (7.9%)	48 (2.2%)

### Medical and psychiatric history

Medical, psychiatric and SUD prevalence surveyed in the three studies was tabulated in Table 12. Schizophrenia and psychotic disorders were rare, approximately 2% of the sample. Prevalence of major depressive disorder diagnosis was 30%. Bipolar disorder prevalence was surprisingly high at 11% overall and was high even in the study with the lowest rate CTN-0030 (6%). Anxiety disorder prevalence was 34% overall, 12% had a history of clinically significant neurological damage, and 4% of the sample had a seizure disorder. Of note, a large proportion of individuals in CTN-0030 reported significant neurological damage (18%). Overall SUD diagnoses in the past year varied depending on the study (Table 13) with a higher prevalence of alcohol use disorder in CTN-0051 (28% vs. 23% in CTN-0027, p = 0.030), amphetamine (19% vs. 11% in CTN-0027, p<0.001), cannabis use disorder (29% vs. 20% in CTN-0027, p<0.001) and cocaine use disorder (31% vs. 33% in CTN-0027, p = 0.470). Three psychiatric measures were assessed on the ASI questionnaire, as well as in the medical examination. The agreement between the two sources was high for schizophrenia (95%) and but not high for assessments of depression and anxiety (65%).

**Table 12:** Overall prevalence of harmonizable medical and psychiatric Co-morbidities.

	CTN-0027 (N = 1269)	CTN-0030 (N = 653)	CTN-0051 (N = 570)	Overall (N = 2492)
**History of Schizophrenia**				
Yes	32 (2.5%)	8 (1.2%)	7 (1.2%)	47 (1.9%)
No	1235 (97.3%)	645 (98.8%)	563 (98.8%)	2443 (98.0%)
Missing/Not Answered	2 (0.2%)	0 (0%)	0 (0%)	2 (0.1%)
**History of Major Depression**				
Yes	352 (27.7%)	215 (32.9%)	179 (31.4%)	746 (29.9%)
No	917 (72.3%)	438 (67.1%)	391 (68.6%)	1746 (70.1%)
Missing/Not Answered	0 (0%)	0 (0%)	0 (0%)	0 (0%)
**History of Bipolar Disorder**				
Yes	147 (11.6%)	37 (5.7%)	79 (13.9%)	263 (10.6%)
No	1121 (88.3%)	615 (94.2%)	491 (86.1%)	2227 (89.4%)
Missing/Not Answered	1 (0.1%)	1 (0.2%)	0 (0%)	2 (0.1%)
**History of Anxiety/Panic Disorder**				
Yes	382 (30.1%)	204 (31.2%)	257 (45.1%)	843 (33.8%)
No	886 (69.8%)	448 (68.6%)	313 (54.9%)	1647 (66.1%)
Missing/Not Answered	1 (0.1%)	1 (0.2%)	0 (0%)	2 (0.1%)
**History of Neurological Damage**				
Yes	115 (9.1%)	119 (18.2%)	69 (12.1%)	303 (12.2%)
No	1151 (90.7%)	534 (81.8%)	501 (87.9%)	2186 (87.7%)
Missing/Not Answered	3 (0.2%)	0 (0%)	0 (0%)	3 (0.1%)
**History of Epilepsy**				
Yes	40 (3.2%)	22 (3.4%)	49 (8.6%)	111 (4.5%)
No	1229 (96.8%)	631 (96.6%)	521 (91.4%)	2381 (95.5%)
Missing/Not Answered	0 (0%)	0 (0%)	0 (0%)	0 (0%)
**ASI-Lite Depression**				
Yes	97 (7.6%)	291 (44.6%)	333 (58.4%)	721 (28.9%)
No	65 (5.1%)	360 (55.1%)	235 (41.2%)	660 (26.5%)
Missing/Not Answered/Not Administered	1107 (87.2%)	2 (0.3%)	2 (0.4%)	1111 (44.6%)
**ASI-Lite Anxiety**				
Yes	96 (7.6%)	317 (48.5%)	385 (67.5%)	798 (32.0%)
No	66 (5.2%)	335 (51.3%)	185 (32.5%)	586 (23.5%)
Missing/Not Answered/Not Administered	1107 (87.2%)	1 (0.2%)	0 (0%)	1108 (44.5%)
**ASI-Lite Schizophrenia**				
Yes	16 (1.3%)	11 (1.7%)	34 (6.0%)	61 (2.4%)
No	146 (11.5%)	641 (98.2%)	536 (94.0%)	1323 (53.1%)
Missing/Not Answered/Not Administered	1107 (87.2%)	1 (0.2%)	0 (0%)	1108 (44.5%)

**Table 13:** Prevalence of substance use disorder diagnoses past year.

	CTN-0027 (N = 1269)	CTN-0030 (N = 653)	CTN-0051 (N = 570)	Overall (N = 2492)
**DSM Alcohol Diagnosis**				
Yes	251 (19.8%)	0 (0%)	159 (27.9%)	410 (16.5%)
No	843 (66.4%)	0 (0%)	411 (72.1%)	1254 (50.3%)
Missing/Not Answered	175 (13.8%)	653 (100%)	0 (0%)	828 (33.2%)
**DSM Amphetamine Diagnosis**				
Yes	120 (9.5%)	0 (0%)	106 (18.6%)	226 (9.1%)
No	974 (76.8%)	0 (0%)	464 (81.4%)	1438 (57.7%)
Missing/Not Answered	175 (13.8%)	653 (100%)	0 (0%)	828 (33.2%)
**DSM Cannabis Diagnosis**				
Yes	223 (17.6%)	0 (0%)	163 (28.6%)	386 (15.5%)
No	871 (68.6%)	0 (0%)	407 (71.4%)	1278 (51.3%)
Missing/Not Answered	175 (13.8%)	653 (100%)	0 (0%)	828 (33.2%)
**DSM Cocaine Diagnosis**				
Yes	357 (28.1%)	0 (0%)	175 (30.7%)	532 (21.3%)
No	737 (58.1%)	0 (0%)	394 (69.1%)	1131 (45.4%)
Missing/Not Answered	175 (13.8%)	653 (100%)	1 (0.2%)	829 (33.3%)
**DSM Sedatives Diagnosis**				
Yes	153 (12.1%)	0 (0%)	153 (26.8%)	306 (12.3%)
No	941 (74.2%)	0 (0%)	417 (73.2%)	1358 (54.5%)
Missing/Not Answered	175 (13.8%)	653 (100%)	0 (0%)	828 (33.2%)

### Quality of life measures

There were marked differences (p<0.001) in the amounts of pain experienced by the respondents, with approximately three times as many people in CTN-0027 and CTN-0030 reporting severe pain compared to CTN-0051 (see Table 14). CTN-0027, which did not have exclusion criteria for baseline pain management, reported a pattern very similar to CTN-0030 which had the strongest exclusion criteria.

**Table 14:** Baseline pain by trial.

	CTN-0027 (N = 1260)	CTN-0030 (N = 653)	CTN-0051 (N = 570)	Overall (N = 2483)
Pain				
No Pain	193 (15.3%)	121 (18.5%)	235 (41.2%)	549 (22.1%)
Very mild to Moderate Pain	844 (67.0%)	428 (65.5%)	309 (54.2%)	1581 (63.7%)
Severe Pain	210 (16.7%)	104 (15.9%)	26 (4.6%)	340 (13.7%)
Missing	13 (1.0%)	0 (0%)	0 (0%)	13 (0.5%)

### Survey of risky behaviors

As can be seen in Tables 15 and 16, while there are notable differences in the patterns of drug use reported on the RBS, polysubstance drug use was common across all studies in the 30 days leading up to treatment. In general, cocaine was frequently used in CTN-0027 and CTN-0051 and was used by the vast majority in CTN-0051. A large majority of people in CTN-0027 and CTN-0051 used heroin alone, but it was relatively rarely used in CTN-0030. While the prevalence was lower for speedball use, the same overall pattern across the trials was observed. Practically everyone in CTN-0030 was using prescription opioids nearly every day. Prescription opioid use was also widespread in CTN-0027 and CTN-0051. Amphetamine use was relatively rare compared to opioids but was still not uncommon, particularly in CTN-0030.

**Table 15:** Drug use reported in the risk behavior survey by trial.

	CTN-0027 (N = 1269)	CTN-0030 (N = 653)	CTN-0051 (N = 568)	Overall (N = 2490)
**Ever Used Cocaine**				
Yes	539 (42.5%)	486 (74.4%)	253 (44.5%)	1278 (51.3%)
No	730 (57.5%)	167 (25.6%)	315 (55.5%)	1212 (48.7%)
**Cocaine in Last 30 Days**				
Mean (SD)	3.28 (6.62)	0.446 (1.80)	4.40 (7.69)	2.77 (6.21)
Median [Min, Max]	0 [0, 30.0]	0 [0, 20.0]	0 [0, 30.0]	0 [0, 30.0]
Missing	113 (8.9%)	0 (0%)	0 (0%)	113 (4.5%)
**Ever Used Heroin**				
Yes	1080 (85.1%)	142 (21.7%)	496 (87.3%)	1718 (69.0%)
No	189 (14.9%)	511 (78.3%)	72 (12.7%)	772 (31.0%)
**Heroin in Last 30 Days**				
Mean (SD)	24.6 (9.52)	0.126 (0.562)	21.7 (11.8)	17.2 (13.8)
Median [Min, Max]	30.0 [0, 30.0]	0 [0, 6.00]	30.0 [0, 30.0]	25.0 [0, 30.0]
Missing	116 (9.1%)	0 (0%)	0 (0%)	116 (4.7%)
**Ever Used Speedball**				
Yes	169 (13.3%)	24 (3.7%)	109 (19.2%)	302 (12.1%)
No	1100 (86.7%)	629 (96.3%)	459 (80.8%)	2188 (87.9%)
**Speedball in Last 30 Days**				
Mean (SD)	1.81 (5.62)	0.00153 (0.0391)	1.96 (5.70)	1.24 (4.67)
Median [Min, Max]	0 [0, 30.0]	0 [0, 1.00]	0 [0, 30.0]	0 [0, 30.0]
Missing	575 (45.3%)	0 (0%)	0 (0%)	575 (23.1%)
**Ever Used Opioids**				
Yes	557 (43.9%)	652 (99.8%)	280 (49.3%)	1489 (59.8%)
No	712 (56.1%)	1 (0.2%)	288 (50.7%)	1001 (40.2%)
**Opioids in Last 30 Days**				
Mean (SD)	7.84 (11.2)	28.5 (3.35)	7.06 (10.7)	13.7 (13.4)
Median [Min, Max]	1.00 [0, 30.0]	30.0 [0, 30.0]	0 [0, 30.0]	7.00 [0, 30.0]
Missing	273 (21.5%)	0 (0%)	0 (0%)	273 (11.0%)
**Ever Used Amphetamines**				
Yes	197 (15.5%)	253 (38.7%)	132 (23.2%)	582 (23.4%)
No	1072 (84.5%)	400 (61.3%)	436 (76.8%)	1908 (76.6%)
**Amphetamines in Last 30 Days**				
Mean (SD)	1.17 (3.38)	0.204 (1.60)	2.10 (5.54)	1.12 (3.82)
Median [Min, Max]	0 [0, 30.0]	0 [0, 30.0]	0 [0, 30.0]	0 [0, 30.0]
Missing	540 (42.6%)	0 (0%)	0 (0%)	540 (21.7%)

**Table 16:** IV drug use describes the most used drug by trial.

	CTN-0027 (N = 1269)	CTN-0030 (N = 653)	CTN-0051 (N = 570)	Overall (N = 2492)
**Days of IV Drug Use in Last 30 Days**				
Mean (SD)	18.3 (13.5)	0.322 (2.65)	16.8 (13.6)	13.2 (14.0)
Median [Min, Max]	27.0 [0, 30.0]	0 [0, 30.0]	14.0 [0, 30.0]	4.00 [0, 30.0]
Missing	0 (0%)	0 (0%)	1 (0.2%)	1 (0.0%)
**Number of Drug Use Events**				
Mean (SD)	2.65 (3.51)	0.0689 (0.522)	1.00 (0)	1.60 (2.76)
Median [Min, Max]	2.00 [0, 60.0]	0 [0, 8.00]	1.00 [1.00, 1.00]	1.00 [0, 60.0]
Missing	0 (0%)	0 (0%)	1 (0.2%)	1 (0.0%)
**Monthly Use Events**				
Mean (SD)	71.4 (101)	1.03 (9.99)	16.8 (13.6)	40.5 (79.0)
Median [Min, Max]	60.0 [0, 1800]	0 [0, 150]	14.0 [0, 30.0]	4.00 [0, 1800]
Missing	0 (0%)	0 (0%)	1 (0.2%)	1 (0.0%)
**Needle Sharing**				
No	1094 (86.2%)	652 (99.8%)	467 (81.9%)	2213 (88.8%)
Yes	175 (13.8%)	1 (0.2%)	102 (17.9%)	278 (11.2%)
Missing	0 (0%)	0 (0%)	1 (0.2%)	1 (0.0%)
**Days Injecting Cocaine**				
Mean (SD)	2.33 (3.45)	1.01 (0.0953)	2.29 (3.41)	2.19 (3.29)
Median [Min, Max]	1.00 [1.00, 22.0]	1.00 [1.00, 2.00]	1.00 [1.00, 22.0]	1.00 [1.00, 22.0]
Missing	731 (57.6%)	543 (83.2%)	2 (0.4%)	1276 (51.2%)
**Days Injecting Heroin**				
Mean (SD)	22.1 (12.3)	1.00 (0)	17.1 (13.7)	19.9 (13.2)
Median [Min, Max]	31.0 [1.00, 31.0]	1.00 [1.00, 1.00]	15.0 [1.00, 31.0]	30.0 [1.00, 31.0]
Missing	190 (15.0%)	613 (93.9%)	2 (0.4%)	805 (32.3%)
**Days Injecting Speedballs**				
Mean (SD)	6.64 (6.73)	1.00 (NA)	2.61 (4.28)	3.53 (5.23)
Median [Min, Max]	3.00 [1.00, 22.0]	1.00 [1.00, 1.00]	1.00 [1.00, 22.0]	1.00 [1.00, 22.0]
Missing	1100 (86.7%)	652 (99.8%)	2 (0.4%)	1754 (70.4%)
**Days Injecting Opioid**				
Mean (SD)	1.58 (2.55)	1.25 (1.94)	1.87 (3.17)	1.55 (2.59)
Median [Min, Max]	1.00 [1.00, 22.0]	1.00 [1.00, 22.0]	1.00 [1.00, 22.0]	1.00 [1.00, 22.0]
Missing	713 (56.2%)	5 (0.8%)	4 (0.7%)	722 (29.0%)
**Days Injecting Stimulants**				
Mean (SD)	2.17 (2.07)	1.00 (0)	1.62 (1.95)	1.72 (1.95)
Median [Min, Max]	1.00 [1.00, 13.0]	1.00 [1.00, 1.00]	1.00 [1.00, 13.0]	1.00 [1.00, 13.0]
Missing	1072 (84.5%)	615 (94.2%)	2 (0.4%)	1689 (67.8%)

There are notable differences in the patterns of injection drug use reported on the risky behavioral surveys (Table 16). Overall, injection drug use was common in CTN-0027 (69%) and CTN-0051 (70%), but rare in CTN-0030 (2.5%), likely due to injection heroin use being a study exclusion criterion. Sharing needles was also common. Injection of cocaine was common for CTN-0027 and CTN-0051, and a small minority of individuals also reported injection of amphetamines (for CTN-0027 and for CTN-0051).

IV drug use was most evident in CTN-0027, both in terms of the number of substances and of drug use events. CTN-0051 showed slightly less daily drug use and fewer drugs used per day, and IV drug use was largely absent in CTN-0030.

### Randomization

Figs 1–3 summarize the number of people screened and randomized per trial. Like most clinical trials, these studies gathered demographic screening data on many people who were not randomized (N = 1,068). This “extra” sample includes 45 people who provided TLFB data useful for describing polysubstance patterns before treatment.

### Treatment and medication doses

We harmonized the total prescribed medications per the dose log (Table 17). The median duration of the dose was longer for methadone compared to buprenorphine, and buprenorphine dosing was longer in CTN-0051 compared to CTN-0027 or CTN-0030 (combining both Phase 1 and Phase 2) but shorter in CTN-0030 (combining both Phase 1 and Phase 2) compared to CTN-0027 or CTN-0051. The median number of doses of XR-NTX was four. The average daily dose in the first week of treatment for methadone and buprenorphine was eight mg across all three studies. Throughout the course of treatment, the average per-person daily dose of methadone was 73 mg. For buprenorphine, it was 19 mg in CTN-0027, 13 mg in CTN-0030, and 16 mg in CTN-0051. The median maximum daily dose for methadone was 90 mg, and for buprenorphine was 24 for CTN-0027 and 16 for CTN-0030 and CTN-0051 throughout the study. The intervals between the injections were reasonably consistent in CTN-0051 at 28 days.

**Table 17:** Doses of medication administered.

	CTN-0027		CTN-0030	CTN-0051	
	Buprenorphine (N = 727)	Methadone (N = 520)	Buprenorphine (N = 651)	Buprenorphine (N = 270)	Naltrexone (N = 204)
**Number of Doses Throughout the Study**					
Median (Q1—Q3)	90 (20–160)	160 (130–170)	74 (28–140)	120 (48–170)	4.0 (2.0–6.0)
**Length of Treatment (in Days)**					
Median (Q1—Q3)	130 (25–170)	170 (160–170)	92 (27–150)	130 (54–170)	100 (21–140)
**Average Dose Throughout the Study**					
Median (Q1—Q3)	19 (14–25)	73 (54–96)	13 (10–16)	16 (12–19)	1.0 (1.0–1.0)
**Maximum Dose Throughout the Study**					
Median (Q1—Q3)	24 (16–32)	90 (70–110)	16 (14–24)	16 (12–20)	1.0 (1.0–1.0)
**Doses in the First Week of Medication**					
Median (Q1—Q3)	8.0 (6.0–8.0)	8.0 (7.0–8.0)	8.0 (8.0–8.0)	8.0 (8.0–8.0)	1.0 (1.0–1.0)
**Average Daily Dose in the First Week of Medication**					
Median (Q1—Q3)	16 (11–21)	45 (39–53)	15 (11–15)	12 (8.0–16)	1.0 (1.0–1.0)
**Maximum Daily Dose in the First Week of Medication**					
Median (Q1—Q3)	16 (13–24)	50 (40–60)	16 (12–18)	12 (8.0–16)	1.0 (1.0–1.0)

## Discussion

In this study, we report the creation of the largest individual-level, comprehensive dataset for OUD from the harmonized databases of three NIDA CTN studies. The resulting dataset, anonymized and released into the public domain with complete documentation, represents a new resource for future investigators to develop new statistics and data science projects to ask specific questions in all three studies with relative ease. Ongoing work with these harmonized data is allowing the development of new data science tools and the evaluation of study-specific treatment effects for subpopulations in these trials. Further work is assessing whether similar patterns are seen in other CTN trials. Our results also yield the broadest view to date of the types of patients who enter pragmatic clinical trials of treatments for OUD and therefore, afford an approximation of the real-world settings they were recruited from.

It *is* important to reflect on the problems with generalization from clinical trials to the application of findings to real-world settings [56]. No clinical trial can encompass the day-to-day care for people battling addiction and it has long been suspected that people in trials obtain benefits compared to people not in trials [57] It is noteworthy that the three harmonized studies described here are pragmatic trials. This means that they assessed the application of the most prescribed medications used to treat OUD, in real world settings, and the studies were designed with relatively broad inclusion criteria. Their success as pragmatic trials is evident in the diversity of the people studied and the extent of the comorbid conditions we document. Conversations with the principal investigators, reviewing the work captured here, indicate that no one fully appreciated the breadth of the diversity of the patients that the three studies collectively covered. Nevertheless, users of this harmonized data should be reminded that the participants in these trials were volunteers who were seeking treatment and are therefore not a representative sample of individuals who use substances.

On the methodological side, our project created a uniform way to construct substance use self-reports from heterogeneously obtained TLFB and UDS data. We discovered that in general, substantial domain expertise was necessary to interpret the instruments contained in the publicly available datasets, which presents a barrier for cross-disciplinary collaborative efforts. More specifically, our reorganization of the data allows for standardized derived concepts (e.g., “UDS-Morphine”) to create databases from underlying data streams from several different studies.

Our proposed reorganization affords the ability to use a Common Data Model (CDM) for SUD clinical trials. This structure will allow investigators to borrow strengths across trials by easily using the tools for visualizing, summarizing, and modeling data that the CTN-0094 team is developing. Converting clinical trial data into this proposed CDM also dictates that investigators resolve issues involving dates and randomization in the design stage, and as discussed below, points to the need to build aggressive data quality checks into their data collection entry systems.

Another methodological note is that in future studies of sequential randomization, there should always be one “primary” randomization after patients are checked for eligibility. Using this date, all data can be systematically “sanity checked” to prevent the entry of dates in the future or before the study time window. Finally, the resulting database is very similar to several standard CDMs, such as the OMOP CDM [58].

While clinical trials in the United States typically enroll only approximately 50 people, thus possibly allowing researchers the ability to hand-curate data, the cleaning of avoidable problematic data at any scale, prior to harmonization, is a waste of valuable resources [59]. The cleaning and harmonization of these data afforded us the opportunity to study how clinical data are gathered, stored, and shared. Two PhD level data scientists/statistical programmers with decades of experience spent many hundreds, if not thousands, of hours cleaning these data. While it is widely acknowledged that data cleaning and preprocessing before modeling takes the majority of analysts’ time (60–80% is a widely quoted percentage), we suggest that a large proportion of this work could be avoided if relatively simple changes were made to data collection systems [60]. Future data cleaning and harmonization projects should use tools to automatically log time-on-task to allow policy makers to understand the real costs of cleaning and harmonizing data.

The issues we noted in the methods section offer us the ability to make suggestions for future studies. The lack of consistent, complete documentation in a computer-readable format proved to be a major obstacle to our work. We observed numerous deviations from the principles for database design described by [61] at the start of the relational database era and reiterated in the R community as tidy data principles [62]. We discovered a plethora of data entry errors in the publicly released datasets from each of these clinical trials during harmonization. Future projects should implement logic checks as part of the data collection systems to prevent problematic data from being entered into research databases in the first place, rather than waiting for issues to be discovered, as we did, after the data lock. We note many cases where data organization was suboptimal for data curation and analysis. These issues and suggested solutions for future studies are enumerated in (Table 18).

**Table 18:** Recommendations.

	Example	Solution
**Issues with Variables**		
Different names for the same concept (like visit subject ID)	tcPATNUM, txtPInumber, PATNUM, ID, PROJID, patientnumber, PATID	Standardize to a subject identifier like “**who**”
Different names for the same concept (like visit date)	VISITDT, VSDTAE, HAMASMDT	Use a common name in all tables, like “**when**”
Variable names not consistently capitalized	txtPInumber, ID	Use a capitalization standard like camel case or **snake case**
Binary variable should not have ambiguous names	sex	Yes/No variables should be named with a leading verb (like **is_male**) and be coded 1 or 0
Study visit indicators must be checked	The visit marked “Week 3” is dated in six weeks after randomization	Add logic checks for *during data collection*
Study dates must be checked	Drug screening results cannot be in the future or before study start	Add logic checks for *during data collection*
Free text must be avoided	Seven spellings of ecstasy	Innumerate likely options as checkboxes *use other as a last resort*
**Issues with Database Organization**		
The same information is in multiple variables	Variables holding: “Heroin use day 1”, “Heroin use day 2”, …, Heroin use day 7”	*Use normalized/tidy/narrow tables* like “**who**”, “**what**” = Heroin, and “**when**” holding the day number
The same information repeated	The UDS data for “Week 6” is repeated as “End of Study (EOS) Record”	Add a status **indicator variables** (like **is_end_of_study**)
**Issues with Documentation**		
Documentation needs to be computer readable	PDFs are difficult for computers to parse	*Document as text* and convert to pretty format
Documentation needs include permitted values	Documentation had permitted values for categorical variables or variables names printed off the edge of a PDF	Categorical variable documentation needs to *explain all values*
Documentation all tables in the database	A questionnaire dropped from a study because of time constraints	*Document all data*

Limitations of the study include the fact that many concepts that we wished to harmonize were missing due to study design related issues. For example, as noted above, not all studies quantified the concept of “heavy drinking”, and other concepts like “sensation seeking behaviors” were not assessed at all. While we created a new CDM that can feasibly receive clinical trial data as an input, this CDM still needs to be cross-referenced with other standardized informatics vocabulary systems such as SNOMED [63] and will require further institutional support to allow it to be used by a community of substance use informatics researchers. Finally, we applied our harmonization strategy specifically to clinical trial datasets, which are comparatively well organized compared to EMR systems, where critical information is frequently nestled in the body of unstructured text notes. A future direction would be to apply the same strategy to more extensive observational databases such as EMR and claims databases that have similar results but with more complex structures.

While our goal was not to develop and test new methods of harmonization, this study’s use of a “double helix” approach to harmonization, with two independent data science teams working to prepare data, proved invaluable because it afforded us two opportunities to identify, and then resolve, myriad problems. Future harmonization studies, perhaps using synthetic data which include the kinds of errors we identified, could quantify the probability that harmonized data is, in fact, clean when it is processed by a single team or two teams working independently, as we described here.

In conclusion, while our primary goal was to harmonize the data to allow for the prediction of treatment success, these harmonized data will save analysts countless hours by eliminating the need to reclean free text responses, correct mistaken drug treatment administration records, deal with inconsistent, multiple UDS records created by date typos, and deal with inconsistencies when a “study week” entry did not reflect reality. Further, the use of the widely accepted Tidyverse Style Guide for table and variable names will lighten analysts’ memory load by eliminating inconsistent naming conventions and data values. Additionally, the release of documentation in a format that both computers and analysts can easily use will allow these data to be merged into subsequent harmonization projects. More importantly, these data support myriad tasks. Currently, biostatistics classes at the University of Miami are using these data to teach data processing and machine learning. The R/Medicine 2024 conference has used these data for a competition to see who can produce the best statistical graphic, summary table, and prediction of treatment success/failure. Ongoing work by the CTN-0094 team is exploring race and ethnicity biases in the dozens of published definitions of treatment success and failure [64]. None of this work could have been undertaken without these harmonized data. Researchers should use these data to improve our ability to predict who responds best to various forms of treatment. Further, they should explore subgroup effects in people seeking care for OUD that would otherwise not be discoverable and/or testable in smaller, unharmonized datasets.
